# Autophagy: A Key Player in Pancreatic Cancer Progression and a Potential Drug Target

**DOI:** 10.3390/cancers14143528

**Published:** 2022-07-20

**Authors:** Josef Gillson, Yomna S. Abd El-Aziz, Lionel Y. W. Leck, Patric J. Jansson, Nick Pavlakis, Jaswinder S. Samra, Anubhav Mittal, Sumit Sahni

**Affiliations:** 1 Faculty of Medicine and Health, University of Sydney, Camperdown, Sydney, NSW 2050, Australia; jgil9462@uni.sydney.edu.au (J.G.); ysal2128@uni.sydney.edu.au (Y.S.A.E.-A.); llec6616@uni.sydney.edu.au (L.Y.W.L.); patric.jansson@sydney.edu.au (P.J.J.); nick.pavlakis@sydney.edu.au (N.P.); jas.samra@bigpond.com (J.S.S.); anubhav@mittal.com.au (A.M.); 2 Bill Walsh Translational Cancer Research Laboratory, Kolling Institute of Medical Research, St Leonards, Sydney, NSW 2065, Australia; 3 Oral Pathology Department, Faculty of Dentistry, Tanta University, Tanta 31527, Egypt; 4 Cancer Drug Resistance and Stem Cell Program, University of Sydney, Sydney, NSW 2006, Australia; 5 Upper GI Surgical Unit, Royal North Shore Hospital and North Shore Private Hospital, St Leonards, Sydney, NSW 2065, Australia; 6 Australian Pancreatic Centre, St Leonards, Sydney, NSW 2065, Australia; 7 School of Medicine, University of Notre Dame, Darlinghurst, Sydney, NSW 2010, Australia

**Keywords:** pancreatic ductal adenocarcinoma, autophagy, tumor microenvironment, stress, autophagy inhibitors

## Abstract

**Simple Summary:**

With the mortality rate of pancreatic cancer predicted to rise over the coming years, it is essential that effective treatment strategies are developed as soon as possible. Pancreatic cancer has always proven very difficult to treat due to its fast growing and aggressive nature. Chemotherapeutic treatment has struggled to increase the survival rate of pancreatic cancer patients due to effective chemo-resistant properties that derive from the supporting tumor microenvironment and autophagy, a vital survival pathway. This review will explore how the autophagy pathway and tumor microenvironment help to sustain tumor survival under stress and expand into a metastatic state. Due to the comprehensive understanding of the autophagy pathway, we will highlight the potential chinks in the pancreatic tumor’s armor and identify potential targets to overcome chemo-resistance in pancreatic cancer. We will also present novel autophagy inhibitors that could reduce tumor survival and how they could be most effectively conceived.

**Abstract:**

Pancreatic cancer is known to have the lowest survival outcomes among all major cancers, and unfortunately, this has only been marginally improved over last four decades. The innate characteristics of pancreatic cancer include an aggressive and fast-growing nature from powerful driver mutations, a highly defensive tumor microenvironment and the upregulation of advantageous survival pathways such as autophagy. Autophagy involves targeted degradation of proteins and organelles to provide a secondary source of cellular supplies to maintain cell growth. Elevated autophagic activity in pancreatic cancer is recognized as a major survival pathway as it provides a plethora of support for tumors by supplying vital resources, maintaining tumour survival under the stressful microenvironment and promoting other pathways involved in tumour progression and metastasis. The combination of these features is unique to pancreatic cancer and present significant resistance to chemotherapeutic strategies, thus, indicating a need for further investigation into therapies targeting this crucial pathway. This review will outline the autophagy pathway and its regulation, in addition to the genetic landscape and tumor microenvironment that contribute to pancreatic cancer severity. Moreover, this review will also discuss the mechanisms of novel therapeutic strategies that inhibit autophagy and how they could be used to suppress tumor progression.

## 1. Introduction

In 2020, pancreatic cancers (PC) had the 14th highest incidence of cancer and placed 7th for the most deaths at a mortality rate of approximately 94% [1]. When compared to the cancers with more deaths per year (i.e., lung, breast, liver, stomach, colon, esophagus) PC have the worst prognostic outcomes among all major cancers [1]. Notably, the mortality rate of PC has only marginally decreased since 2000 [2] and is considered one of the deadliest types of cancer. PC are more prevalent in western populations, which is likely due to the rise of non-genetic risk factors such as smoking, alcohol consumption, pancreatitis, obesity and, subsequently, diabetes [1,3]. In fact, PC is predicted to become the third highest cause of cancer-related death in Europe by 2025 [4]. The most common subtype of PC are pancreatic ductal adenocarcinomas (PDAC) which occur within the ductal acinar cells in the exocrine compartment of the pancreas and account for over 90% of incidences [5,6]. This review will focus on PDAC.

There are many factors associated with PDAC pathology which makes it a challenging clinical disease. The symptoms that PDAC patients exhibit are often non-specific (e.g., abdominal pain, nausea, weight loss, etc.), which makes diagnosis very difficult and often results in advanced, late-stage disease [7]. In addition to this, PDAC has driver mutations such as *Kirsten’s rat sarcoma viral oncogene homologue* (*KRAS*) and *tumor protein p53* (*TP53*) which can synergize to exert a vast range of growth and tumor supporting signals [8,9]. Unfortunately, the biomarker available to aid PDAC detection, i.e., Ca 19-9, lacks specificity and sensitivity at the early stages [10]. Advanced PDAC tumors are also highly resistant to chemotherapy due to a supportive tumor microenvironment (TME) [11,12]. The TME of PDAC is characterized by excessive metabolic stress which stimulates sustained growth potential [13]. Moreover, the TME comprises of a supporting network of fibroblasts which increase extracellular matrix (ECM) density and forms a protective stromal barrier [14]. Recently, there has been an increased appreciation of the critical role played by metabolically and mechanically stressed TME in PDAC progression [15,16].

Autophagy is a versatile, stress responsive, self-degradation process that can target damaged and unwanted proteins and organelles for recycling into smaller and useable metabolic substrates such as amino acids [17,18]. Under normal physiological conditions, autophagy is tightly regulated by a range of influential upstream proteins and pathways that is only executed when necessary or when there is a specific target [18]. Autophagy is a widely interactive pathway that can be regulated by core cellular processes such as transcription, translation, cell cycle, cell signaling, cellular stress and enzymatic pathways [17,19,20]. Due to the diversity of these interactions, autophagy is often utilized by PDAC under stressful TME conditions and is known to contribute to PDAC progression and chemotherapy resistance [21]. As current chemotherapeutics have been ineffective at providing a curative treatment for PDAC, the need to identify new targets has become essential to improve patient treatment outcomes. This review will outline the current relationship between PDAC and autophagy and discuss recent insight into novel autophagy inhibitors which have the potential to repress this complex disease.

## 2. Autophagy

### 2.1. Autophagy Types and Selectivity

The degradation of cellular contents is a central process in all eukaryotic cells [22,23], which is primarily performed by the ubiquitin-proteasomal system (UPS) and autophagy [17,23,24]. The UPS is a highly specialized mechanism that targets old, dysfunctional or unwanted cellular material through ubiquitination and degrades the content into smaller molecular units [17,19,24]. Autophagy features a more versatile targeting spectrum as it can incorporate organelles and a more diverse range of proteins than UPS [18,24,25].

**Figure 1:**
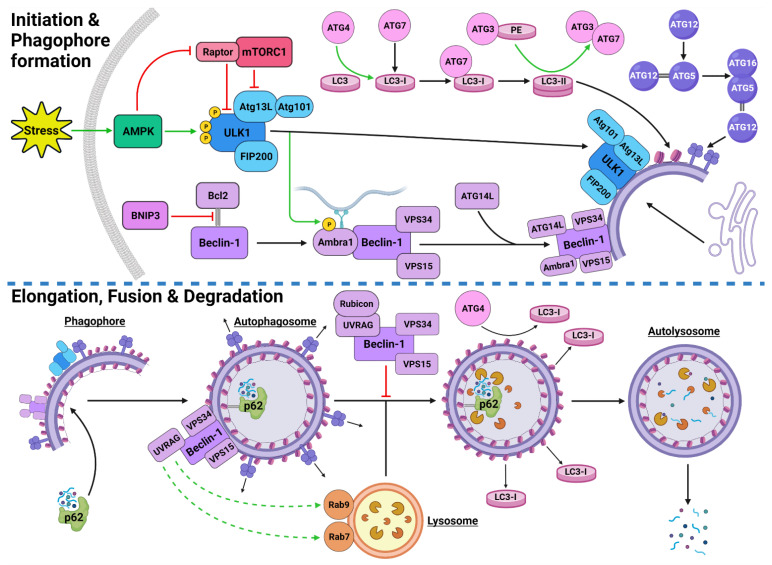
**Stress-Induced Autophagy Pathway and Machinery.** Tumor microenvironmental stress stimulates autophagy via AMPK activation which induces autophagic initiation. The ULK1 complex and PI3KC3-C1 facilitate phagophore formation which matures and elongates into an autophagosome by structural proteins LC3-II and the ATG5-ATG12-ATG16 complex. The autophagosome forms around the target protein/organelle and fuses with a lysosome mediated by the PI3KC3-C2. The cargo is degraded into various biomolecules and released into the cytoplasm. Black arrows indicate binding to or moving to, green arrows indicate activation, green dashed arrows indicate attraction, red arrows indicate inhibition. *Created with BioRender.com (accessed on 24 February 2022)*.

Autophagic activity can be executed by three main mechanisms: chaperone-mediated autophagy (CMA), microautophagy and macroautophagy [26,27,28]. CMA is a highly specific process and relies on the recognition of unique targeting motifs located on certain cytosolic substrates by a cytoplasmic chaperone, such as Hsc70, which leads to their delivery to lysosomes for degradation [29,30]. Microautophagy possesses both specific and non-specific targeting and involves the direct invagination of targets into lysosomes [31]. The mechanism underlying macroautophagy is characterized by the *de novo* formation of phagophores around cytoplasmic structural mass, which matures into an autophagosome that fuses with a lysosome to allow the localized hydrolases to degrade the target protein or organelle into smaller, useable molecules [18,32,33,34] (Figure 1). Macroautophagy will be the focus of this review and will be referred to throughout as “autophagy”.

Autophagy exhibits both selective and non-selective targeting of cytoplasmic contents [35,36,37,38]. While these mechanisms use the same intracellular core machinery, selective targeting utilizes a range of specialized receptors and chaperones [39]. The autophagic chaperone p62 is known to sequester the targeted protein/organelle and carries it to a receptor on the autophagosome for degradation [38,39]. Additionally, non-selective autophagy of small cytoplasmic proteins is more prominent under normal conditions and during early stages of stressful starvation events [40]. In contrast, prolonged stressful starvation triggers a rise in specific autophagic targeting of more complex proteins and organelles [40]. This indicates that stress can instigate a stronger, more selective response. Some examples of autophagic selectivity include pexophagy (peroxisomes) [41], mitophagy (mitochondria) [42,43] and xenophagy (bacteria during an infection) [44,45].

The process of autophagy occurs in all cell types and is an integral part of homeostatic regulation throughout the cellular lifecycle [46]. However, autophagy is well established as a stress-responsive process that is highly upregulated during starved conditions to generate more energy and nutrients [47,48,49], cellular remodeling from growth and development [50,51], and increased during oxidative stress [37,52].

### 2.2. Autophagy Process and Machinery

#### 2.2.1. Autophagy Initiation

The initiation of autophagic flux is regulated by two important protein complexes, namely, UNC-51-like kinase (ULK1) complex and phosphoinositide 3-kinase class III-complex 1 (PI3KC3-C1) (Figure 1). When phosphorylated by its upstream regulators, ULK1 can bind to both the focal adhesion kinase family interacting protein of 200 kDa (FIP200) and the conjugate of autophagy-related protein 13 (ATG13) and ATG101 to form the ULK1-FIP200-ATG13-ATG101 complex, which is also known as the ULK1 initiation complex [53,54]. The ULK1 initiation complex is vital for the completion of autophagy and its inhibition was shown to significantly reduce autophagic initiation and prevent cell survival under nutrient-deprived conditions [55,56]. Upon its formation, the ULK1 initiation complex triggers an array of downstream signaling pathways to begin the formation of an isolation membrane, known as a phagophore [57]. The most significant of these signals involves the activation of PI3KC3-C1 [57,58]. This ULK1-mediated phosphorylation of Beclin-1 can be enhanced by both ATG14-like (ATG14L) and ultraviolet radiation resistance-associated gene protein (UVRAG) [57].

**Figure 2:**
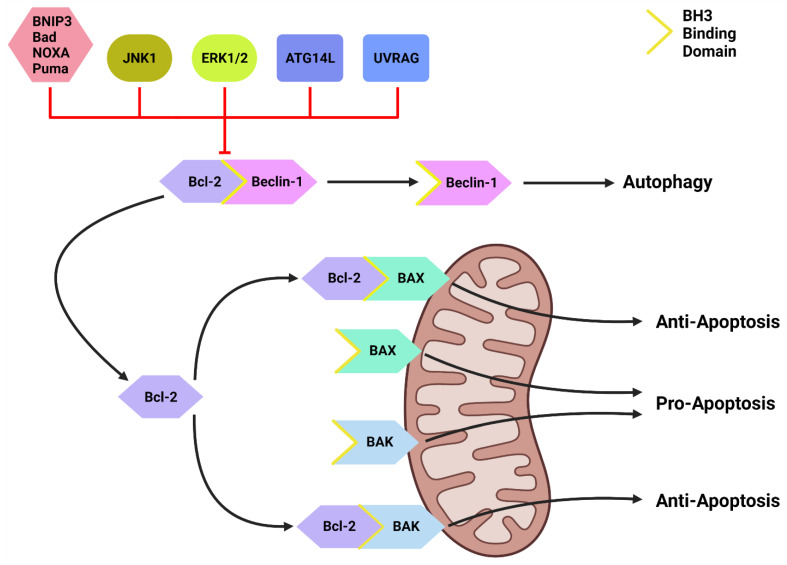
**The Bcl-2/Beclin-1 Interaction.** Bcl-2 family proteins (BNIP3, Bad, NOXA, Puma) that compete for the BH3 binding site and other proteins such as JNK1, ATG14L and UVRAG can disrupt the Bcl-2/Beclin-1 complex. This disruption frees Beclin-1 to form the PI3KC-C1 and initiate autophagic initiation or PI3KC-C2 to promote autolysosome fusion. Free Bcl-2 can also bind to the BH3-binding site on BAX and BAK to protect the mitochondria and suppress apoptotic function. *Created with BioRender.com (accessed on 4 March 2022)*.

Inactive Beclin-1 is bound to B-cell lymphoma 2 (Bcl-2) [59] (Figure 2). When released by other competitive BH-3-binding proteins, such as BCL2/adenovirus E1B 19 kDa protein-interacting protein 3 (BNIP3) (Figure 2), Beclin-1 binds to vacuolar protein sorting 34 (VPS34), VPS15 and, autophagy and Beclin-1 regulator 1 (AMBRA1), which anchors the complex to microtubular dynein [58,60] (Figure 1). ULK1 activates the PI3KC3-C1 by: **(1)** phosphorylating AMBRA1 to release it from the dynein; and **(2)** phosphorylating both Beclin-1 and VPS34 allowing ATG14L to bind [58,61,62,63]. The activated ULK1 complex and PI3KC3-C1 then localize to the isolation membrane on the ER/golgi apparatus [64,65].

#### 2.2.2. Autophagosome Formation

The PI3KC3-C1 and ULK1 complex facilitate phagophore elongation into an autophagosome, which is characterized by two ubiquitin-like systems, namely, ATG5-ATG12-ATG16 conjugate and microtubule-associated proteins 1A/1B light chain 3A (LC3) [60,66] (Figure 1). ATG5 forms a covalent bond with ATG12 which is later joined by ATG16 [67,68]. LC3 is converted to LC3-I by ATG4, which stimulates the binding of ATG7 to attract ATG3 resulting in the ligation of phosphatidylethanolamine (PE) with LC3-I to form LC3-PE conjugate (i.e., LC3-II) [32,69,70]. A multitude of LC3-II and ATG5-ATG12-ATG16 complexes then localise to the phagophore to begin the formation of the autophagosome [68] (Figure 1).

#### 2.2.3. Autophagosome Maturation

The maturation process depends upon interactions between LC3-II and sequesterome-1 (p62), which are also two distinct markers of autophagic flux [71] (Figure 1). LC3 is one of three human homologs of Atg8 in yeast, the other two are GABARAP and GATE-16, both of which function similar to LC3 [72]. Similarly, p62 shares its role with the homologs BNIP3L, NBR1 and Alfy [73,74,75]. p62 provides selectivity to the autophagic process by recognizing ubiquitinated target proteins and sequestering the target towards the phagophore [18,73]. It then binds directly to the internal membrane-bound LC3-II using the LC3 recognitions sequence in a ligand-receptor-like manner, which then stabilizes the target protein in place to allow the autophagosome to form around it [39,76]. Upon autophagosome formation, external LC3-II remains on the surface of the membrane, the internal LC3-II and p62 are enclosed within the membrane, while the ATG5-ATG12-ATG16 complex progressively detaches [77] (Figure 1). Interestingly, *ATG3* knockouts generated autophagosomes lacking LC3-II that were still able to bind to p62 and complete autophagic flux, suggesting that the ATG5-ATG12-ATG16 complex was able to attract p62 independently of LC3-II [39]. This result supports the concept that autophagy is a tightly regulated process with complex machinery that can adapt and respond to various forms of disruption throughout the process.

#### 2.2.4. Autolysosome Formation and Cargo Degradation

Once autophagosomes have fully enveloped their target, they fuse with lysosomes to form autolysosomes [78]. Interestingly, UVRAG competes with ATG14L for the same binding site on Beclin-1 [62]. With UVRAG bound, this complex is known as the PI3KC3-C2 and facilitates the attraction of the lysosome-bound GTPases, Rab7 and Rab9 to the autophagosome [62,79,80,81] (Figure 1). Rubicon can bind to UVRAG to mediate a suppressive effect on autolysosome formation through the interference with Rab7 attraction [82,83]. However, it was demonstrated that in circumstances of sustained autophagic activation, the UVRAG expression levels outnumber the Rubicon expression, and therefore, manages to maintain dominance of Rab7 activation and trigger autolysosome formation [83].

Upon autolysosome formation, the acidic hydrolases and proteases from the lysosome target the contents and remaining membranous proteins which causes proteolysis to yield smaller products such as amino acids, peptides and free fatty acids [84] (Figure 1). These products are released into the cytoplasm to be reused, excreted into the bloodstream for use elsewhere, restore the intracellular free amino acid pool or directly transported to the ribosome for protein synthesis [84,85]. The degradation of internally bound LC3-II and p62 is indicative of autophagic flux [86]. The externally bound LC3-II is not degraded, but delipidated by ATG4 into LC3-I, which can then be reused in the next autophagic cycle [72].

### 2.3. Upstream Autophagy Regulation

Autophagy regulation upstream of the core autophagic machinery involves numerous proteins and pathways that indirectly activate or inhibit this critical catabolic process. The major upstream pathways involved in regulation of autophagic machinery are: **(i)** PI3K class I (PI3KC1)/protein kinase B (AKT)/mammalian target of rapamycin complex 1 (mTORC1) pathway; **(ii)** mitogen activated protein kinase (MAPK) pathway; **(iii)** adenosine monophosphate-activated protein kinase (AMPK); and **(iv)** Bcl-2.

#### 2.3.1. PI3K/AKT/mTORC1 Pathway

**Figure 3:**
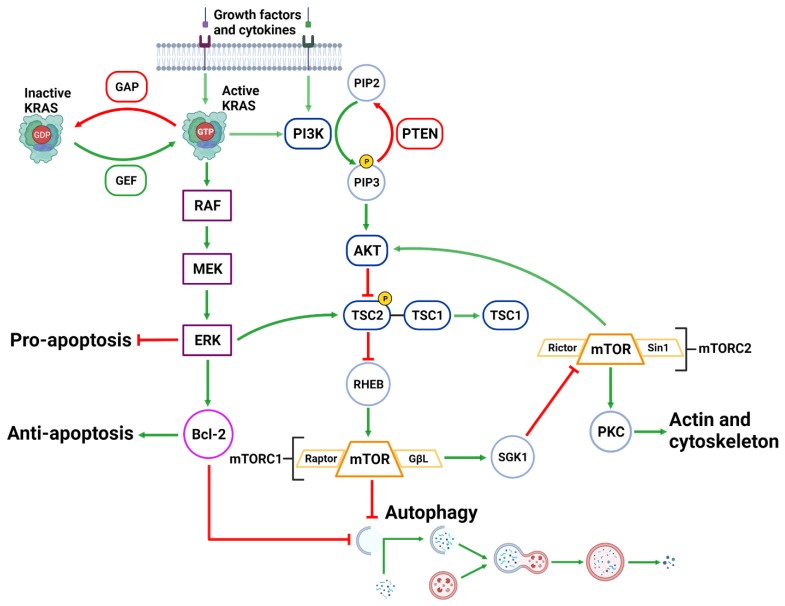
**Upstream Autophagy Regulation.** Extracellular growth factors and cytokines activate KRAS and PI3K. GAP and GEF regulate KRAS activity which begins the MAPK cascade of activating RAF, MEK and ERK. ERK can inhibit pro-apoptotic function and support anti-apoptotic function via Bcl-2. ERK can also inhibit mTORC1 which facilitates autophagic initiation. PI3K phosphorylates PIP3 which is regulated by PTEN dephosphorylation. PIP3 activates AKT causing the destabilization of TSC2-TSC1 complex. This supports mTORC1 activity and suppresses autophagic initiation. mTORC1 can also regulate AKT via a feedback loop and suppress cytoskeleton activity involving SGK1 and mTORC2. *Created with BioRender.com (accessed on 28 March 2022)*.

The activation of the PI3KC1 complex from either receptor tyrosine kinases or KRAS leads to constitutive phosphorylation of the phospholipid PIP2 into PIP3 [87] (Figure 3). Phosphatase and tensin homolog (PTEN) functions to reverse the action of PI3K by directly dephosphorylating PIP3 back into inactive PIP2 to maintain regulation of the pathway [88,89]. PIP3 activates AKT, which then phosphorylates tuberous sclerosis complex 2 (TSC2) at five different sites (Ser939, Ser981, Ser1130, Ser1132 and Thr1462) causing it to destabilize and dissociate from TSC1 [90,91]. This dissociation prevents the dual protein complex of TSC1 and TSC2 from inhibiting ras homolog enriched in brain (RHEB), a constitutive activator of mTORC1 [90]. mTORC1 is comprised of mTOR, GβL, PRAS40 and Raptor [92]. Sustained mTORC1 activity mediates autophagy suppression via the phosphorylation of the major initiation proteins ULK1 (Ser757) and ATG13L, rendering them inactive [53,54,93,94,95]. Therefore, the activation of PI3K/AKT/mTORC1 inhibits autophagy while the suppression of the PI3K/AKT/mTORC1 pathway promotes autophagy [87,96] (Figure 3). Additionally, mTORC1 operates to activate S6K1 and destabilizes the eIF-4E and 4E-BP1 complex to collectively promote protein synthesis [87,97], further reinforcing its significance in managing cellular protein synthesis or degradation.

#### 2.3.2. MAPK Pathway

Downstream of KRAS, the rapid accelerated fibrosarcoma (RAF)/mitogen-activated protein kinase kinase (MEK)/extracellular signal-regulated kinase (ERK) pathway operates parallel to the PI3K/AKT pathway and is similarly integral to growth signaling with a major influence on tumor onset and survival [98,99] (Figure 3). In addition to transcription-related nuclear effects and the regulation of cytosolic proteins [100], MEK and ERK1/2 are also known for their crosstalk into other pathways which allows for an increased range of effects on autophagy when compared to the PI3K/AKT pathway. This can be epitomized by ERK1/2 activation of TSC2 (at the ERK D domain) which leads to RHEB inhibition and subsequent mTORC1 destabilization [101] (Figure 3). This results in increased levels of Beclin-1 and ULK1 leading to significantly increased autophagic initiation [101,102]. Additionally, phosphorylation of Bcl-2 by ERK1/2 is shown to promote its dissociation from Beclin-1, resulting in autophagic induction [103] (Figure 2).

Interestingly, the strength of the MEK/ERK signal dictates the effectiveness of autophagy activity, such that moderate MEK/ERK activity-induced cyto-protective autophagy and sustained MEK/ERK activation can cause cyto-destructive autophagy [101]. ERK inhibition or *ERK* knockdown was unable to fully repress autophagic flux [101]. However, MEK inhibition was found to completely abrogate autophagic activity [101]. As ERK is one of the downstream MEK effector proteins, this result indicates that MEK was capable of bypassing ERK and used alternative effector proteins to sustain the stimulatory autophagic signal.

The effects of MAPK activity may be described as a more versatile and passive signaling pathway than a binary pathway. In liver and breast cancer, MEK inhibition (PD98059) completely suppressed rapamycin and serum starvation-induced autophagy observed from sustained mTORC1 activity and reduced Beclin-1 levels [101]. It has therefore been described that MEK/ERK activation is required for autophagy activation [104]. Interestingly, there is also evidence of a feedback network where autophagy related genes are capable of regulating ERK1/2 phosphorylation. Either *MAP1LC3* or *ATG5* mRNA silencing in mice resulted in reduced ERK phosphorylation and suppressed MAPK signaling [105].

#### 2.3.3. AMPK

**Figure 4:**
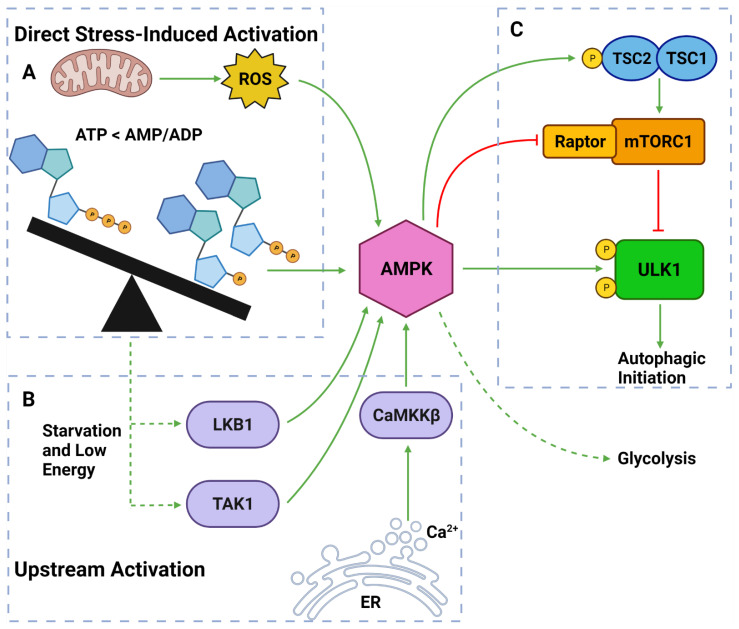
AMPK Regulators and Effectors. (**A**) AMPK can be directly activated by stress from: overworked/stressed mitochondria produced ROS; AMP and ADP due to decreased ATP levels. (**B**) AMPK can also be activated by upstream regulators: LKB1 and TAK1 which respond to reduced energy levels; CaMKKβ which responds to increased cytoplasmic calcium from ER stress. (**C**) Once activated, AMPK can induce autophagy by: phosphorylating TSC2; inhibiting Raptor on mTORC1; phosphorylating ULK1 at Ser317 and Ser777. Activated AMPK can also upregulate glycolytic activity. *Created with BioRender.com (accessed on 6 April 2022)*.

AMPK is a well-established upstream regulator of autophagy [54,71,106]. As a stress-responsive protein, AMPK reacts to decreased cellular energy and resource levels to stimulate survival pathways such as autophagy and glycolysis [106,107] (Figure 4). AMPK consists of a regulatory γ subunit, a structural β subunit and a catalytic α subunit [108]. Stress-associated AMPK activation occurs from a direct mechanism involving depleted adenosine triphosphate (ATP) levels that raise cytoplasmic adenosine mono/diphosphate (AMP/ADP) levels [109,110] (Figure 4). AMPK is also activated by three upstream regulators in response to different stimuli: **(1)** liver kinase B1 (LKB1), which responds to cellular energy levels; **(2)** Ca^2+^/calmodulin-dependent kinase kinase β (CaMKKß) activation by increased cytoplasmic calcium (Ca^2+^) from ER stress; and **(3)** transforming growth factor-β (TGF-β)-activated kinase 1 (TAK1) [106,107,111,112] [113,114] (Figure 4).

Once activated, AMPK upregulates autophagy via from 3 major pathways: **(1)** phosphorylation and deactivation of Raptor (a protein within the mTORC1); **(2)** activation of TSC2, causing RHEB inhibition and subsequent mTORC1 inhibition; and **(3)** ULK1 phosphorylation at Ser317 and Ser777 sites [53,54,94,115] (Figure 4). It should also be noted that the activation of TSC2 can directly oppose the PI3K pathway-induced autophagic suppression [106,107,111]. Collectively, AMPK is a vital autophagic activator which has a complex, yet well understood, mechanism of activating autophagy.

#### 2.3.4. Beclin-1 & Bcl-2

Another important regulator of autophagic initiation involves the Bcl-2 family of apoptosis-related proteins. The Bcl-2 protein itself typically exerts anti-apoptotic signaling where it binds to BH3 domains on pro-apoptotic proteins such as Bcl-2-associated X protein (BAX) and Bcl-2 homologous antagonist killer (BAK) to protect the mitochondria [116] (Figure 2). Beclin-1 also contains a BH3 binding domain and has been found bound to Bcl-2 in the form of a dual protein complex [59,117,118]. Importantly, the Beclin-1/Bcl-2 complex restrains Beclin-1 from initiating autophagy and prevents Bcl-2 from binding to pro-apoptotic proteins, leading to increased apoptosis [59,117,118] (Figure 2).

Beclin-1 can be dissociated from Bcl-2 via **(1)** JNK1; **(2)** other BH3 domain containing Bcl-2 family members, such as BNIP3, Bad, Noxa, Puma, etc.; and **(3)** other autophagy promoting proteins such as UVRAG and ATG14L [119,120] (Figure 2). This suggests that Bcl-2 plays a major role in the crosstalk between apoptotic and autophagic machinery.

## 3. Pancreatic Cancer

### 3.1. Current Treatment Options

PDAC is known as a “silent killer” due to its limited symptom presentation and biomarker availability which often leads to diagnosis at advanced stages [10,121,122,123]. Surgery is the only current treatment option that is intended to potentially cure PDAC, but only ~20% of patients have resectable disease at the time of diagnosis [124]. Even for resectable patients, surgery is often not a standalone treatment and may require neoadjuvant chemotherapy prior to resection or adjuvant chemotherapy afterwards [122,124]. Due to innate PDAC chemoresistance, the concept of combination therapy has become more viable than monotherapy. FOLFIRINOX is a novel chemotherapeutic strategy composed of fluorouracil, leucovorin, irinotecan and oxaliplatin [125]. When administered to patients post-operatively, this heavily loaded treatment regime increased survival by 14.8% and only featured mild higher toxicity compared to gemcitabine alone [125]. The clinical trial by Conroy et al., involving 342 metastatic patients demonstrated superior overall survival and objective response with FOLFIRINOX over gemcitabine [126]. The future of PDAC treatment needs to: **(1)** involve more reliable and measurable biomarkers to detect PDAC at earlier stages; **(2)** more effectively predict the patient treatment plan; and **(3)** continue the discovery of more efficacious compounds that provide a significant increase in survival rate without the expense of toxic adverse effects.

### 3.2. Genetic Landscape of PDAC

PDAC develops from an accumulation of certain inherited and acquired mutations in the cellular DNA. There are four major driver mutations for PDAC progression, namely, *KRAS, tumor protein 53* (*TP53*)*, SMAD Family Member 4* (*SMAD4*) and *cyclin dependent kinase inhibitor 2A* (*CDKN2A*).

#### 3.2.1. KRAS

Overactivation mutations of the *KRAS* gene are observed in ~30% of all cancers and is widely considered to be responsible for neoplastic transformation and a major driver for tumor proliferation [127,128]. The *Ras* gene family is made up of three homologs: *KRAS*, *HRAS* and *NRAS* [129]. In PDAC, *KRAS* is the most frequently mutated gene, with mutations occurring in codon 12 and 13 (70–90%) and the most common being *KRAS^G12D^* (>50%) [9,130]. This high rate of *KRAS* mutations has also been strongly linked to poor PDAC prognosis [9]. Under normal conditions, KRAS is a well-connected membrane bound GTPase that is activated through the exchange of GDP to GTP and is regulated by GTPase activating protein (GAP) [131,132,133,134] (Figure 3). KRAS transmits its signals to downstream targets in the PI3K, ERK, NFĸB and mTOR pathways, which are responsible for cell survival, proliferation and cytokine production [135,136] (Figure 3). Mutated *KRAS* results in a continuously activated GTP-bound state where the GAP deactivation protein is deemed obsolete and essentially allows the persistent binding of effector proteins, such as PI3K and RAF, to bind and transmit the growth signals downstream to the nucleus to perpetually stimulate tumor growth [131,134,137]. The upstream initiators of KRAS are growth factors such as epithelial growth factor (EGF) and insulin-like growth factor (IGF) which are known to promote cellular growth, protein synthesis and proliferation [97]. In tandem with *KRAS*, mutations in *AKT1* (~50%), *AKT2* (10–20%), *PI3KC1* (5%) and the regulator protein *PTEN* (>70%) are also common in PDAC [138,139,140]. These genes are components of a highly influential pathway capable of inducing a variety of tumor supporting actions, including autophagy. Downstream KRAS activity via the MAPK pathway is demonstrated to stimulate autophagy, while its effect on PI3K/AKT pathway results in inhibition of autophagic activity [89,141]. Overall, there is a complex role played by KRAS in autophagic regulation in PDAC, as discussed above in Section 2.3.

#### 3.2.2. TP53

*TP53* is also frequently mutated in PDAC (22%) and synergizes with *KRAS* mutations to increase the chance of metastasis in PDAC patients (50–75% in PDAC when *KRAS* mutations are present) [8,142]. *TP53* mutations are often characterized by an accumulation rather than deletion or loss of the protein where mutant *p53^R172H^* involves gain of function or dominant-negative properties [8]. p53 is a cellular reactor to DNA damage, oncogene activation and other cancer related stressors such as hypoxia and oxidative stress [8]. When activated, p53 can exert its tumor suppressive properties through both transcriptional and non-transcriptional mechanisms that induce autophagy, cell-cycle arrest, senescence and, in severe cases, apoptosis to terminate irreparable cells [143]. Additionally, tumors that feature *TP53* mutations will lack the ability to trigger apoptosis in damaged cells and hence, allow tumor progression [143]. In fact, one study identified that all grade 3 PDAC tumors featured *TP53* mutations suggesting a strong link between p53 and advanced PDAC [144]. Additionally, mice featuring *p53^R172H^* mutations were able to progress into metastatic PDAC, while p53 deficient mice were not [8]. This result distinguishes the mutant *TP53* from *TP53* knockout or deficiency.

The relationship between autophagy and p53 is intricate and involves many different interactions. Autophagy sustains survival by self-degradation while p53 can trigger apoptosis [145]. A genomic profile of p53 highlighted its role in autophagy activation as it transcribes a range of genes involved in the core autophagic machinery (*ATG7*, *ATG10*, *ULK1* and *UVRAG*) [143]. Additionally, p53 is known to promote autophagy, and upon autophagic completion, p53 activity is suppressed [145]. This indicates that p53 and autophagy maintain a homeostatic balance to determine whether the cell can repair itself or requires apoptosis. Interestingly, nuclear p53 stimulates autophagy via the expression of specific genes (e.g., *ULK1* and *ATG7*), whereas cytoplasmic p53 inhibits autophagy, indicating that the subcellular localization of p53 drives its functionality [146].

#### 3.2.3. SMAD4

*SMAD4* is commonly deleted or negatively mutated in PDAC patients (40-60%) and results in a shorter median survival [147,148,149]. *SMAD4* operates as a tumor suppressor of the epithelial-mesenchymal transition (EMT)-promoting transforming growth factor-β (TGF-β) signaling pathway and has the capacity to induce apoptosis [150,151]. Interestingly, TGF-β1 was able to induce autophagy using both SMAD4-dependent and independent mechanisms. In PDAC with SMAD4 present, TGF-β1-induced autophagy supported proliferation and suppressed migration [152]. Whereas, SMAD4-deficient PDAC cells demonstrated reduced proliferation and increased migration [152]. This observation suggests that SMAD4 presence determines the outcome of TGF-β1-induced autophagy and that PDAC-deficient in SMAD4 may have accelerated EMT and higher metastatic potential.

#### 3.2.4. CDKN2A

The two tumor suppressor proteins transcribed by *CDKN2A*, p14 and p16, operate as cell cycle regulation proteins by preventing progression through G1 and G2 checkpoints [153]. If *CDKN2A* is negatively mutated in PDAC (30–50%) [8,148], these proteins are improperly transcribed, leading to uncontrolled neoplastic proliferation [154]. p14 has been found to prevent UPS-mediated p53 degradation via MDM2, which can subsequently lead to autophagy induction [155]. Additionally, p16 was found to induce autophagy in cancer associated fibroblasts (CAFs) [153]. Interestingly, this means that PDAC with *CDKN2A* mutations may feature reduced autophagy but a significantly increased growth rate.

#### 3.2.5. PTEN and BRCA1/2

Negative mutations in the tumor suppressor genes *PTEN* (70%) and breast cancer gene 1/2 (*BRCA1/2)* (5–20%) are detected in later stages of PDAC as they provide auxiliary support to progress the tumors into a more malignant state [139,147,149]. Mutated *PTEN* involves a negative mutation or deletion that restricts the normal regulation of the PI3K/AKT pathway [156,157] (Figure 3). *PTEN* deletion does not cause tumor formation in pancreatic β-cells and is not considered a major driver of PDAC [158]. However, they are highly synergistic with *KRAS* or *SMAD4* mutations and can lead to severe prognosis [159,160,161]. Mice with *KRAS^G12D^, PTEN^wt/−^* and mutations of autophagic machinery proteins demonstrated a significantly lower survival more than mice without mutations in autophagic proteins [161]. However, with *KRAS^G12D^* and total *PTEN^−/−^* deletion, there was no difference in survival between mice with or without mutations in autophagic proteins [161]. Through the suppression of the PI3K/AKT pathway, PTEN activity supports the impact of autophagy on tumor formation and development via oxidative stress, altered metabolism, inflammation and DNA damage [162]. Collectively, these studies suggest that PTEN is capable of influencing autophagy both dependently and independently of the PI3K/AKT/mTOR pathway and is a crucial anti-tumor protein.

Germline *BRCA1/2* mutations account mostly for familial PDAC [163]. BRCA1/2 operate in the protection of the genome through DNA repair and homologous recombination respectively [164]. While a significant driver mutation in breast cancer, BRCA1/2 mutations have recently received more attention in PDAC research. This is due to its importance in repairing single stranded DNA damage by poly-ADP ribose polymerase (PARP). Therefore, PARP inhibition can induce double-stranded DNA damage which cannot be properly repaired by *BRCA1/2* mutant PDAC cells, leading to cell death [165]. The clinical trial by Golan et al. measured the effectiveness of the PARP inhibitor, olaparib, in PDAC patients with germline *BRCA1* or *BRCA2* mutations [166]. They found that progression-free survival was significantly longer in those treated with olaparib compared to placebo patients [166]. While this study was comparing single-drug therapy to placebo, the results suggest that olaparib could benefit from non-DNA targeting combination therapy, such as autophagy inhibition. There is little research on the effect of autophagy in *BRCA1/2*-mutated PDAC. However, in triple negative breast cancer a novel autophagy inhibitor (SBP-7455) demonstrated significant synergy with the PARP inhibitor, olaparib [167]. Considering tumors deficient in *BRCA1/2* are highly sensitive to PARP inhibitors [168,169], this result suggests a potential relationship between BRCA1/2 and autophagy that requires more investigation.

## 4. Pancreatic Tumor Microenvironment and Autophagy

### 4.1. Tumor Microenvironment

A tumor is a highly complex mass of various cells that is driven by mutations associated with unrestricted growth and cytoprotective abilities to promote tumor progression [170]. Even though each tumor is unique to its organ of origin, the extensive review by Hanahan and Weinburg identified six hallmarks of cancer which characterize the main features of all tumors [170]. These hallmarks can be described as: **(1)** sustained proliferative signaling; **(2)** evasion of growth suppressors; **(3)** activation of invasion and metastasis; **(4)** immortality; **(5)** induction of angiogenesis; **(6)** immunosurveillance evasion; and **(7)** resistance to programmed cell death [170]. These hallmarks are key features required for tumor progression into an advanced and metastatic state. The initial cell, or group of cells, that undergo neoplastic transformation cannot grow to a sizeable mass and attain metastasis without the aid of surrounding cells [15]. The combination of metabolic and mechanical changes experienced by aggressive tumors feature detrimental stressful conditions that are alleviated through locally recruited supporting cells and the activation of various survival pathways [171,172]. It is these supporting cells along with conditions in the cancerous tissue surrounding the tumor mass that constitute the TME.

### 4.2. Pancreatic Tumor Microenvironmental Stress

As aggressive neoplasms have increased proliferation rates, they require an higher demand for nutrients and resources from the blood supply [170]. Such tumors, such as advanced PDAC, cannot receive adequate blood flow as they typically outgrow their local blood supply and become overly dense from the stromal layer which reduces blood vessel penetration [173,174]. Pancreatic tumors suffer from a considerable reduction in blood volume and flow when compared with healthy pancreatic tissue [175]. This leads to areas of necrotic tissue and stressed cells in an uninhabitable microenvironment featuring ischemia, hypoxia, acidosis and low energy [173,175,176,177]. Multiple studies have found that an increasing distance from blood vessels reduces proliferation rates and increases ischemia and necrosis [178]. The cells in the core of tumor tissue are further nutrient deprived and rely more on survival pathways to sustain life or are pressured to migrate to a safer environment [177]. Mammalian cells have evolved to counter these harsh conditions by activating numerous pathways to make resources available, such as anaerobic respiration, angiogenesis and autophagy [56,84,179]. The presence of the stressful microenvironment around tumor cells forces them to adapt to survive.

#### 4.2.1. Altered Energy Metabolism

The cells most valuable resource, ATP, is chemically synthesized from glucose by anaerobic and aerobic respiration [180,181]. It is vital for tumors as they rely on this energy source to grow. However, the rapid proliferation and restricted blood supply of aggressive tumors such as PDAC exhausts local oxygen and ATP availability. Therefore, aerobic respiration, involving oxidative phosphorylation, is often limited [180,182,183]. In order to meet the demanding energy needs of tumors, glycolysis is upregulated even under oxygen-sufficient conditions (aerobic glycolysis), a phenomena which is known as the Warburg effect and is typically observed in PDAC [13,184]. This pathway is highly inefficient due to low ATP yield and produces excessive hydrogen ions which creates a hyper-acidic cellular environment [185,186,187].

AMPK responds to low ATP levels using a homeostatic mechanism that involves the maintenance of glycolysis through the upregulation of glycolytic metabolism [188] (Figure 4). PDAC cells treated with the AMPK inhibitor, compound C, resulted in impaired aerobic glycolysis, cell death via apoptosis and reduced metastatic potential [188]. This result reinforces the importance of aerobic glycolysis in the progression of aggressive cancers such as PDAC. Recently, challenges with gemcitabine therapy in PDAC were ascribed to the induction of metabolic reprogramming from oxidative phosphorylation towards aerobic glycolysis [189]. This was identified as a result of gemcitabine-induced AMPK activation and the direct activation of KRAS which promotes cancer stemness and tumor progression [189]. Notably, combination treatment of gemcitabine with an aerobic glycolysis inhibitor (i.e., 2-deoxy-D-glucose) demonstrated a significantly enhanced anti-cancer activity in PDAC cells [189].

The activation of AMPK increases both autophagic flux and aerobic glycolysis [93,188] (Figure 4). It is strongly established that AMPK activity helps maintain tumor survival by inducing autophagy in response to a stressful TME [93,106,190,191]. Therefore, highly stressed PDAC cells, especially those within the solid core, that endure major metabolic deficits are equipped with the ability to balance the expenditure and production of ATP through the upregulation of autophagy and aerobic glycolysis [13,192].

#### 4.2.2. Reactive Oxygen Species (ROS)

While PDAC metabolic reprogramming upregulates glycolysis, tumor cells still rely on the significant ATP yield produced by the tricarboxylic acid (TCA) cycle within the overworking mitochondria [193]. The main sources of ROS in cancer are derived from increased cellular metabolism mechanisms including increased NADPH oxidase (NOX) activity in the mitochondria and glycolysis from the Warburg effect [194,195,196,197,198,199]. Highly stressed PDAC cells with increased energy demands upregulate these pathways and therefore, produce a larger amount of ROS [184,199]. ROS are detoxified by antioxidant enzymes such as superoxide dismutase and catalase and are typically expressed at higher rates in PDAC [194,195,196,197,198,200]. However, the significantly increased ROS levels overwhelm these antioxidant defense pathways. The detrimental effects from ROS include DNA damage, lipid peroxidation and protein oxidation [194,195,201,202], which allows ROS to possess oncogenic capabilities and play a critical role in tumor initiation and progression [199]. Additionally, ROS within the mitochondria can inhibit Bcl-2 and allow BAX and BAK to subsequently induce apoptosis via the caspase pathway [203] (Figure 2). This pathway is often treated as a self-killing response if the cell becomes uninhabitable from overloaded ROS. Therefore, low to medium levels of ROS are tumor-supporting whereas excessive amounts can be cyto-destructive.

PDAC tumor cells featuring *KRAS*, *MAPK* or *PI3K/AKT* pathway mutations are often overstimulated [130,140,159]. ROS produced by overworking mitochondria is deemed essential for KRAS-mediated cell growth [202]. These ROS have been found to directly activate the pro-growth and anti-apoptotic proteins ERK1/2 and AKT, and indirectly promote NFĸB-mediated apoptosis evasion [172,195,202,204]. If damaged cells manage to evade systematic repair or cell-mediated apoptosis, they are at risk of developing cancerous mutations and transitioning into tumor cells [198]. This evasion is one of the hallmarks of cancer [170]. ROS also promote the EMT and neoplastic migration through the activation of hypoxia inducible factor 1α (HIF-1α) and NFĸB [205,206]. In addition to HIF-1α-induced Beclin-1 activation [205], both HIF-1α and NFĸB also promote the expression of major EMT-associated transcription factors Snail, Slug, Twist1 and ZEB1/2 [206], indicating a strong relationship between ROS and tumor progression.

It is well documented that ROS can activate autophagy [207,208]. One of the major factors regulating this is AMPK, which is activated by ROS and starvation conditions [207] (Figure 4). When ROS generation was blocked, AMPK activation was suppressed leading to repressed ULK1 activity and increased inhibitory regulation of autophagy by mTORC1 [207,209]. Moreover, ROS have been demonstrated to increase autophagic activity independently of AMPK [209,210]. This has been reported due to increased conversion of LC3 to LC3-I via a thiol modification of the Cys81 site on ATG4 and, subsequently, increased autophagosome accumulation [211]. ROS are also shown to be involved in the disruption of Beclin-1 and Bcl-2 to facilitate Beclin-1 induced autophagic initiation [209].

In PDAC cells, gemcitabine-induced ROS was able to increase KRAS activity [189]. KRAS has been found to activate AMPK, which can support the Warburg effect, and subsequently lead to increased glycolysis and autophagy upregulation [189]. Therefore, this study provides a link between increased ROS and autophagy induction in PDAC. Notably, the addition of an autophagy inhibitor (i.e., chloroquine) to gemcitabine treatment was reported to significantly enhance gemcitabine-mediated apoptosis [212].

ROS saturation and potential cytotoxicity can be ameliorated in stressed PDAC through the upregulation of autophagy and mitophagy to recycle damaged mitochondria which can prolong tumor survival and increase the likelihood of metastasis [209]. Additionally, autophagy upregulation in PDAC has also been found to mitigate ROS levels and reduce subsequent DNA damage, suggesting an adaptive homeostatic mechanism to sustain tumor survival under stressed conditions [213].

#### 4.2.3. Acidosis

Specific transporters and exchangers such as NHE-1 and v-ATPase maintain cellular pH homeostasis by releasing acidic molecules into the extracellular environment to maintain a neutral intracellular pH [185,214]. As the tumor-associated blood and lymphatic vessels cannot effectively drain the interstitial fluid due to inconsistent distribution and poor perfusion, the hydrogen ions accumulate to cause a heterogeneous acidic environment and exert numerous effects on the surrounding components of the TME [175,215,216,217]. These cellular mechanisms can influence PDAC within an acidic TME. There is also significant evidence that an acidic TME can maintain autophagic stimulation over extensive periods of time and is capable of sustaining tumor cell survival [218,219,220]. The specific mechanism involving the induction of autophagy under acidic conditions has not been entirely elucidated. However, it has been observed that an acidic environment increased ATG5, BNIP-3 and LC3-II levels, indicating autophagic upregulation [220].

There is evidence of acid-sensing ion channels (ASICs) which internalize hydrogen ions to stimulate autophagy and promote EMT [221]. Interestingly, ASICs are shown to be upregulated in PDAC [221]. Wang et al. found that *ASIC1a* knockdown and PcTx1 (ASIC inhibitor) treatment was able to suppress acid-induced autophagy in pancreatic stellate cells (PSCs) [218]. These findings further highlight the benefit of targeting autophagy in PDAC, considering that autophagy is essential for PSC activation [222], and PSCs are vital for PDAC progression [223,224].

#### 4.2.4. Hypoxia and Angiogenesis

As neoplasms begin to advance beyond a small set of cells, to form a tumor mass, they will require more nutrients and resources from the blood supply [170]. While ischemic conditions can still yield ATP via the Warburg effect, other nutrients obtained from fresh blood flow are required for other cellular function beyond ATP production [170,225]. Tumor cells exercise the upregulation of new blood vessel formation via angiogenesis pathways to maximize nutrients and support tumor proliferation [170,226,227]. This has been confirmed by a large-scale meta-analysis of female breast cancer patients [228] and more recent findings in luminal breast and advanced non-small cell lung cancer [227,229], which demonstrated a high microvessel density to be highly associated with poor prognosis since it provides more nutrient resources for the tumor. PDAC features a thick stromal outer layer that is largely hypovascular due to bulk extracellular matrix [182,183]. This dense stroma is known to significantly reduce blood flow to the tumor [182,183]. This reduced blood flow is a major contributor to PDAC metabolic stress and increases their survivability through the activation of AMPK, HIF-1/2α and the subsequent upregulation of survival pathways such as autophagy.

The two hypoxia inducible factors HIF-1 and HIF-2 are the main proteins involved in the detection and adaptation to reduced oxygen availability [230,231]. Hypoxic conditions reduce the proteasomal degradation of HIF-1/2α and allow the formation of a dimer with HIF-1β, which can then migrate to the nucleus and transcribe relevant genes involved in angiogenesis, EMT, migration and autophagy upregulation [230,232,233,234]. In PDAC, HIF-1/2α become overexpressed and have been strongly associated with advanced clinical staging, metastasis and poor patient prognosis due to the ability to promote survival and apoptotic resistance [235,236,237]. This suggests that PDAC can adapt to the harsh TME and can result in therapeutic resistance.

HIF-associated hypoxic response elements can transcribe the pro-apoptotic protein, BNIP-3, which functions to disrupt the Beclin-1-Bcl-2 complex [232]. The current literature suggests that the liberation of Beclin-1 allows it to form the PI3KC3-C1 and initiate autophagy; and more specifically, mitophagy [232,238,239]. Partial mitophagy has been found to help reduce mitochondrial overload and ROS generation as part of a major survival response initiated by HIF-1α [240]. While BNIP-3 is expressed in hypoxic conditions, it was only able to execute apoptosis under acidic conditions [232]. This result suggests that this mechanism of apoptosis may only be functional in advanced and highly stressed cells such as PDAC.

Of the genes transcribed by HIF-1/2α, 25 out of 70 (e.g., *VEGF*, *PDGF* and *TGF-β*) are involved in angiogenesis stimulation to boost the influx of fresh blood [241]. While it is beneficial for tumor cells to receive more blood for tumor growth, it can also enhance drug delivery deeper into the tumor. As PDAC is often not resectable, the hypo-vascularity in PDAC is one of the major pillars in its chemo-resistance because it significantly reduces the drug perfusion into the tumor cells [241,242]. This forms a double-edged sword hypothesis regarding the potential benefits and problems with angiogenic inhibition in hypo-vascular tumors such as PDAC, especially if combined with cytotoxic chemotherapeutics. The extent of the relationship between angiogenesis and autophagy derives from HIF-1/2 instigating the transcription of autophagy related genes such as *BECN1*, *ATG5* and *ATG7*, and enhancing LC3-I to LC3-II conversion [234,243]. Consequently, angiogenesis inhibition would only be effective at starving the tumor cells of essential nutrients. Therefore, angiogenesis inhibition may not be beneficial in PDAC since it would support hypoxia-induced HIF activity, which can reduce drug effectiveness by actively upregulating angiogenesis in stressful circumstances and promoting pro-tumorigenic effects such as autophagic induction.

#### 4.2.5. Extracellular Matrix and Mechanical Stress

While metabolic stress is focused more on intracellular metabolic pathways involving nutrients and energy, mechanical stress relates to structural components of the TME, extracellular interactions and kinetic forces [15,16]. There has been a shift in the chemotherapeutic approach to PDAC, firstly due to chemoresistance, but also due to observations of a highly dense stromal matrix [14,244]. The stroma in some PDAC tumors can account for up to 90% of the tumor mass [244]. This has led to the development of nanoparticles bound to chemotherapeutics or core capsules that can deliver a nano-bomb of compounds to aid the delivery [245,246]. This protective stroma is fundamental to PDAC survival, aggressive nature and chemotherapeutic resistance. Most of the stromal bulk consists of ECM proteins such as glycoproteins, collagen and elastin which are modified by proteinases and proteases [247,248]. The high density of the ECM encourages stimulatory interactions between neoplastic and supporting TME cells and concentrates the local tumor-promoting cytokines and growth factors [249,250,251]. The uncontrolled deposition and remodeling of the ECM is the fundamental feature to mechanical stress. This creates local tension known as interstitial pressure, which can cause inflammation and fluid buildup within the PDAC stroma [249].

To maximize metastatic potential, PDAC tumors require increased cell motility and a dynamic and manipulated ECM from the supporting PSCs and CAFs. Another important factor in cell invasion is the actual process of breakaway, which involves reduced levels of calcium-dependent epithelial cadherin (E-cadherin) and other proteins that are vital for cell adhesion and contact inhibition [252]. In tumors such as PDAC, the adaptation to mechanical and metabolic stress can be used in tandem to progress metastasis. As the environment in the core of PDAC tumors becomes more uninhabitable from hypoxic, ischemic and acidic conditions, the cells are subjected to migratory and invasion signals which promotes neoplastic breakaway and increases motility through the modified ECM [170,176,185,216,249,253,254]. This ultimately increases the likelihood of tumor cells entering the blood and lymphatic vessels to metastasize [255]. Additionally, the stromal bulk and poor local fluid drainage is a major barrier preventing efficient chemotherapeutic delivery [244].

High stiffness due to the development of a dense stroma has been found to significantly upregulate autophagy using LC3-I/II levels as markers for autophagic flux [256]. This was reported to be the result of markedly increased AMPKα levels, which was confirmed to enhance overall AMPK activity due to the observation of increased acetyl CoA carboxylase activity, an enzyme directly downstream of AMPK [256]. The mechanism underlying this involves detection of increased cell-cell contact and stiffness via integrin αV (ITGAV) receptors on the plasma membrane [256]. ITGAV then promotes internal focal adhesion kinase (FAK) to recruit focal adhesion proteins which stabilize and protect AMPK. Under these high stiffness conditions, this protection leads to a longer half-life and increased longevity and activity of AMPK [256], and therefore, supporting AMPK-induced autophagic stimulation. Intriguingly, LKB1 induced the colocalization of AMPK to E-cadherin in response to increased mechanical stiffness [257]. This interaction occurs in a similar fashion to that of ITGAV and FAK and reinforces the importance that PDAC stromal stiffness has on autophagy.

In PDAC, autophagy has also been identified to degrade E-cadherin. In PDAC models featuring intermittent hypoxic conditions, *HIF1α* siRNA and PI3K inhibition (3-MA) was used to inhibit autophagy [233]. This resulted in increased E-cadherin and a significantly higher number of PDAC cells featuring epithelial morphology [233]. This was also observed in the study involving the combination of ERK1/2 and autophagy inhibition, which identified increased E-cadherin and reduced vimentin expression [258]. Therefore, as autophagy is increased through TME-induced stress, the potential for E-cadherin degradation is more likely and can subsequently promote EMT and metastasis.

### 4.3. Supporting Cells of the Tumor Microenvironment

Within the 3D structure of PDAC tumors the neoplastic cells become surrounded by a supportive complex of recruited cells and protective stroma as the tumor progresses [174,177]. The interactions between the residing and recruited cells of the stroma combined with bulk protein deposition are responsible for driving PDAC into metastasis [14]. Histologically, the main cellular components of the PDAC stroma are cancer-associated fibroblasts (CAFs), pancreatic stellate cells (PSCs), mesenchymal stem cells and infiltrating immune cells such as macrophages [259,260].

#### 4.3.1. Cancer Associated Fibroblasts and Pancreatic Stellate Cells

Under *in vitro* co-culture of both PDAC and CAFs, the metastatic potential of neoplastic cells was significantly increased when compared to PDAC cells alone [222,260]. This was demonstrated to be due to the result of both metabolic and mechanically supporting effects and suggests that impairment of supporting CAFs can be beneficial for slowing tumor progression and metastasis. A good example of TME synergy instigating the recruitment of a supporting network involves neoplastic and local inflammatory and endothelial cells activating resident dormant PSCs through paracrine signaling [14,249,261,262]. Such activating signals include PDGF, TGF-β, SHH, COX-2, TNF-α, IL-1, IL-6, IL-8, TRAIL and ROS [12,249,250,262,263]. Once activated, these PSCs display a pancreas-specific phenotype similar to myofibroblasts and effectively function as CAFs through the modification of the ECM [264,265]. It is also important to note that stellate cells within the liver and pancreas have been described as mesenchymal stem cells since they can replicate their function, such as the induction of GATA1 for blood formation and the support of hematopoietic and progenitor cells [266]. PSCs are equipped with autocrine signaling involving the release and reception of the majority of the aforementioned factors to further enhance tumor growth [12]. This dual regulation acts as another barrier to the chemoresistance as it becomes essential to PSC vitality, meaning they can maintain their active state and encourage metastasis through self-perpetuating autocrine mechanisms [12,248].

Interestingly, CAFs are also recruited to provide metabolic support to PDAC tumor cells [267]. It has recently been demonstrated that activated CAFs favor glycolysis through the production of their own pyruvate, which can be secreted, and encourage its uptake by tumor cells [205,223,260]. Even though lactate has been considered a waste product of glycolysis, pancreatic tumor cells have been found to convert it back into useable pyruvate and subsequently acetyl-CoA to fuel the tricarboxylic acid cycle [260]. It has also been shown that epithelial cancer cells induce aerobic glycolysis, via the Warburg effect, in neighbouring stromal fibroblasts [268].

In addition to their primary function of collagen, laminin and fibronectin deposition in the stroma, PSCs also produce matrix metalloproteinases (MMP) which function to modify the ECM [264] [12,269]. In fact, it was observed that MMP-2 was actively expressed in only 3.5% of normal pancreas samples when compared to much higher rates in PDAC tumor cells (55.2%) and PDAC stroma (79.3%), with similar ratios were observed for MMP-7 and MMP-9 [270]. Similarly to the dual autocrine and paracrine regulation, PSCs can self-regulate their MMP production with the release of direct MMP inhibitors and tissue inhibitor of metalloproteinases (TIMP-1 and TIMP-2) [247].

Abundant nuclear yes-associated protein 1 (YAP-1) is important for PSC activation and tumor-supporting paracrine signaling [271]. In fact, *YAP-1* knockout or inhibition was able to deactivate PSCs and severely reduce tumor proliferation [271]. Additionally, YAP-1 can promote *ATG5* transcription, a key protein in autophagosome formation [272]. YAP-1 can also be targeted for autophagic degradation and has been recognized to mediate a negative feedback mechanism to regulate YAP-1 activity [272]. The authors further suggested that this relationship is a vulnerability in PDAC signaling [272]. Interestingly, the combination of YAP-1 inhibition (verteporfin) and autophagic activation (rapamycin) attenuated tumor growth [272]. This result is surprising and demonstrates a highly complex signaling relationship regulating autophagy in PDAC microenvironment.

Sousa et al. reported an interesting relationship between tumor metabolism and alanine as the result of autophagic completion in PSCs [273]. When co-cultured, PDAC cells were demonstrated to stimulate autophagy in PSCs [273]. Increased autophagic degradation produced an increased amount of free alanine which was then secreted from the PSCs and taken up by PDAC cells [273]. The available alanine acts as an alternate fuel source for the TCA cycle and was able to rescue the inhibited growth potential in nutrient-deprived PDAC cells [273]. This study further establishes the importance of autophagy in supporting PDAC growth in both neoplastic cells and supporting cells of the TME.

#### 4.3.2. Schwann Cells

Further support from the TME involves an interesting relationship between autophagy and invasion in local pancreatic Schwann cells. The PDAC cells were found to promote autophagy in Schwann cells by a paracrine signal pathway involving NGF and ATG7 [274]. The “recruited” Schwann cells then: **(1)** promote aggressive perineural invasion in the PDAC cells; **(2)** migrate towards the tumor; and **(3)** promote neoplastic invasion via chemotaxis towards the nerve [274]. The combination of these outcomes can significantly enhance the aggressiveness and metastatic potential of PDAC cells.

#### 4.3.3. Endothelial Progenitor Cells

One of the key cells that are recruited to the growing tumors are endothelial progenitor cells (EPCs). They typically assist with angiogenesis and act to promote new blood vessel formation and tumor growth [275]. When exposed to an acidic environment, there was a reduction in VEGF and IL-8 excretion activity by EPCs, leading to decreased new blood vessel formation [276]. Interestingly, PDAC cells upregulate ASICs to actively internalize hydrogen ions as a counter mechanism to extracellular acidity [218,221]. This leads to an increase in the pH of the extracellular environment, which promotes EPC function and autophagic activity in tumor cells [218,221,276].

#### 4.3.4. Immune Cell Infiltration

PDAC features a range of local immune infiltrate including leukocytes, neutrophils, B and T lymphocytes, macrophages and myeloid progenitors [277]. These immune cells support the tumor cells via cytokine crosstalk which both exude pro-inflammatory (TNF-α, IL-6 and IL-8) and anti-inflammatory (TGF-β, IL-10) outcomes [278]. The pro-inflammatory cytokines have been found to promote the growth and progression of the tumor into metastasis, whereas the anti-inflammatory permit immune evasion and elicit tumor cell protection [278].

Within T-cell lymphocytes, the ever transforming TME involves the shift from T-helper 1 (Th1) immunophenotype, which are associated with anti-tumor activity and good prognosis, into the T-helper 2 (Th2) immunophenotype, shown to support tumors and feature poor survival [278,279]. Macrophages also demonstrate similar dichotomy of phenotypes and can be activated into M1 and M2 immunophenotypes [280]. The M1 is known as the classical phenotype which involves protection from pathogens and tumors via the secretion of tumor necrosis factor-α (TNF-α) and interleukin-12 (IL-12) [281]. It is also associated with longer survival in PDAC patients and promotes the beneficial Th1 phenotype [282]. However, in advanced PDAC, the alternate M2 phenotype, often referred to as tumor-associated macrophages, are more commonly observed [281]. These macrophages promote Th2 phenotype, and are associated with shorter survival [281,282]. Interestingly, the cytokines secreted by the tumor cells determine the type of phenotype, with M1 activation relying on interferon gamma (IFN γ), TNF-β and toll-like receptor ligands; and M2 activation from CD163, mannose receptor, scavenger receptor A and B1 [280]. In addition to varying activation methods, M2 macrophages secrete an alternate set of cytokines and factors to M1. These include VEGF, epithelial growth factor, TGF-β and IL-10, which collectively elicit local immune suppression and neovascularization [259,281]. TGF-β and IL-10 are often over-excreted due to SMAD4 mutations and M2 activity, causing them to dominate the local immune response through Janus kinase-signal transducer and activator of transcription (JAK-STAT) pathway activation [148,278,283].

Interestingly, Jiang et al. established a novel anti-cancer strategy by concurrent administration of MEK inhibitor (cobimetinib), autophagy inhibitor (hydroxychloroquine) and CD40 agonist (aCD40 mAb) in mice implanted with PDAC cells [284]. This combination increased macrophage and natural killer (NK) cell volume and instigated a phenotypic switch from M2 to M1 macrophages [284]. This occurred due to paracrine signaling modification from an increased bias towards STING/type I IFN pathway activation which increased M1-like gene transcription over the M2-favored genes [284]. It is also well established that tumor-associated macrophages produce high levels of ROS which can directly lead to promoting autophagic activation and EMT [205,206,207,208].

The major histocompatibility complex class I (MHC-I) is expressed within the ER of all nucleated cells and is vital in identifying internal antigens and provoking a CD8+ T-cell to bind and initiate an immune response to target the defect cell [285]. In all types of cancer, CD8+ T-cells can alert the immune system of defected cells that are initiating the neoplastic process and target them for destruction [286]. Interestingly, the acidity of the TME has been shown to inhibit natural killer and T-cell activity and therefore, reduce immunosurveillance [185]. MHC-I has been observed at reduced levels in PDAC as a result of increased autophagic upregulation [287]. The recent study by Yamamoto et al. identified that MHC-I molecules were co-localized more with lysosomes and LC3 than the plasma membrane, indicating autophagic degradation [287]. This could lead to an increased number of tumor cells evading the immune system and increasing the likelihood of tumors progressing to later stages. When orthotopically transplanted into allograft mouse models, autophagic inhibition (chloroquine) increased infiltrating CD8+ T-cell levels and resulted in reduced tumor sizes [287]. This groundbreaking paper pioneered the strong connections between autophagy and immune evasion and needs to be further explored to therapeutically exploit this vulnerability.

Taken together, the current research indicates that the reach of autophagy extends to multiple pillars within the hallmarks of cancer and that its suppression could serve as a highly beneficial strategy for the treatment of PDAC.

## 5. Autophagy in Pancreatic Cancer Progression

There has been emerging evidence of a critical role played by the autophagic pathway in pancreatic cancer progression [213,222]. Notably, previous studies have shown that advanced/high grade PDAC have elevated autophagy when compared to normal pancreas or low grade PDAC [210,288]. This is understandable as protein synthesis is vital for the overstimulated growth and unlocking the metastatic potential of cancers [170]. Overall, increased autophagic upregulation in PDAC could be instigated by a combination of driver mutations and a highly stressful TME [15,147]. Autophagy may support stressed neoplastic cells directly by providing more biomaterials or by influencing alternate pathway to support tumor survival.

### 5.1. Autophagic Regulation in PDAC

As discussed in Section 3.2, autophagy is tightly regulated by a variety of upstream pathways that are often mutated in PDAC. The PI3K/AKT pathway mediates autophagic inhibition, while the MEK/ERK pathway is deemed essential for autophagy activation [87,101,289] (Figure 3). KRAS is at the helm of these varying pathways and is frequently mutated in PDAC [290]. While KRAS influences autophagic regulation, it has opposing downstream effectors. Therefore, the net increase to autophagy in PDAC is determined by a balance between upstream regulatory pathways, TME stress and other stress-related proteins such as AMPK and HIF-1/2, which adopt a more primary role at advanced stages [291].

The relationship between AMPK, autophagy and PDAC progression is an area of active research (discussed in Section 2.3.3 and Section 4.2.3). AMPK is often activated from low cellular ATP levels and is regulated by upstream proteins, such as LKB1 and CaMKKß, and can directly promote autophagic initiation through different mechanisms [108,110,292] (Figure 4). Through these interactions, AMPK-induced autophagy is highly prevalent in stressed PDAC and is considered a fundamental component of PDAC survivability [93,106,190,191] (Figure 1). Similarly to AMPK, HIF-1/2 are activated by hypoxic conditions and can promote autophagy via the transcription of BNIP-3 (discussed in Section 4.2.4) [292]. Increased HIF activity further strengthens the survival abilities of stressed PDAC and encourages EMT and neoplastic migration [234]. The excessive ROS levels in stressed PDAC can be simultaneously damaging and supporting [189,195,201]. ROS can directly stimulate AMPK, mTOR and HIF-1α-mediated autophagy, indicating that PDAC tumors are still able to utilize damaging ROS to aid survival and progress to more advanced stages (discussed in Section 4.2.2) [292,293].

### 5.2. Autophagy Promotes Pancreatic Tumor Progression

It has been established that autophagy acts as a tumor suppressor in early stages of PDAC development through the degradation of oncogenic proteins and resistance to apoptosis [294]. However, as the tumor becomes more advanced, autophagy is recorded at abnormally high levels where it operates as a survival pathway and promotes cell growth [294]. TME-induced stress typically inflicts biological responses that prevents cellular growth [173,175,176,177]. However, autophagic upregulation can help aggressive PDAC adapt to the harsh conditions [210]. One of these mechanisms involves autophagic activity opposing apoptotic activity. As discussed in Section 2.3.4, Beclin-1-mediated autophagic initiation is positively correlated with anti-apoptotic Bcl-2 function [118] (Figure 2). Therefore, as autophagy remains activated, apoptotic activity is reduced. This can potentially result in increased tumor survival under TME stress, which provides tumors more time to grow and metastasize; and could suppress cytotoxic chemotherapies from stimulating apoptosis-induced cell death. PDAC chemotherapy is largely ineffective due to the protective stroma and can be further suppressed if tumor cells are actively opposing apoptosis [118,295]. This interaction could also explain why combination therapy involving autophagy inhibition is highly synergistic [179,246,291].

Importantly, cytoplasmic contents deemed unnecessary for tumor proliferation can be degraded into amino and fatty acids and boost the available pool [85]. Free amino acids can be transported into the ER and ribosomes to produce more vital proteins involved in cellular metabolism and cell division [85,296]. To maintain an increased growth rate, PDAC cells can upregulate glycolysis via the Warburg effect (discussed in Section 4.2.1) [13,225]. This increased activity demands more proteins to execute and is therefore, fueled by autophagic degradation [225]. Moreover, in the TCA cycle glutamine is one of the primary sources of carbon [297]. Notably, autophagy has been described as a major source for intracellular glutamine and hence, can directly support oxidative phosphorylation [298].

Autophagy is essential to PSC and CAF function since it can provide alanine for neighboring tumor cells and enhance the deposition of ECM proteins such as glycoproteins, collagen and elastin; and MMPs which increase the ECM remodeling (discussed in Section 4.2.5 and Section 4.3.1) [261,273]. Increased biomaterial availability can enhance the production of a range of proteins involved in various cellular functions. This could include actin, myosin and other cytoskeletal proteins to increase cell motility and promote cellular breakaway [299]. Autophagy has been observed targeting and degrading MHC-I in PDAC resulting in reduced levels (discussed in Section 4.3.4) [287]. Due to the importance of MHC-I in immune surveillance, this degradation can protect the tumor cells and can lead to uncontrolled tumor growth [287]. A recent study has also shown importance of autophagic induction in Schwann cell could promote perineural invasion, which is one of well-known poor prognostic factor in PDAC progression [274].

Current studies examining the relationship between autophagy and pancreatic cancer progression have shown critical importance of this pathway in tumor progression and its potential to be developed as a key therapeutic target for this aggressive disease. Future studies will offer further insights on the complexity of autophagy regulation, its importance to PDAC survival, and how it may be manipulated to provide a therapeutic advantage over the disease.

### 5.3. The Role of Autophagy in Pancreatic Cancer Metastasis

Cancer metastasis is the main cause of cancer-related death in PDAC and is therefore, a crucial area to be investigated [300,301,302,303]. PDAC is often characterized by its early metastatic features, resistance to anti-cancer therapies and poor prognosis [1,304]. Emerging evidence implies that the role of autophagy in cancer progression is complex, and often multifaceted, as contrasting studies suggest that it can be metastasis-promoting or suppressing depending on the stage of the disease, different tumor types and involves other pathway interactions [305,306].

#### 5.3.1. Autophagy as a Metastasis Promoter in Pancreatic Cancer

Most literature regarding PDAC establishes autophagy at a metastasis promoter. As a stress-induced pathway, it is known for maintaining cell survival and promoting the hallmarks of cancer, including metastasis [170,210]. Autophagy directly promotes metastasis through the degradation of proteins involved in focal adhesion. Paxillin is a binding protein that acts a scaffold for the recruitment of other proteins, such as focal adhesion kinase, and is responsible for binding actin in the cytoskeleton and extracellular integrin to create an anchor between cells and the ECM [307]. Autophagy was shown to degrade paxillin resulting in a reduced structural binding between tumor cells and the ECM, thus increasing neoplastic migration [308,309]. A further study using chloroquine (CQ) treatment in breast cancer models demonstrated a reduced rate of paxillin degradation both *in vitro* and *in vivo* [308]. More recently, this interaction has been confirmed in PDAC using a nano-bomb combination of gemcitabine and CQ. This combination was more effective at inhibiting paxillin degradation and downregulating MMP-2 when compared with either mono-treatment [246]. These results in both pancreatic and breast cancer models demonstrate that the autophagic degradation of paxillin led to increased metastatic potential.

Hypoxia-induced autophagy is prominent in PDAC due to the advanced and stressed nature of the neoplasm (discussed in Section 4.2). Intermittent hypoxia was not only shown to upregulate autophagy-related proteins (Beclin-1 and LC3-II), but also increased EMT-related markers (vimentin and N-cadherin) and reduced the level of the cell-to-cell adhering protein, E-cadherin [233]. These latter findings were demonstrated to be due to the induction of hypoxia-induced autophagy [233]. In another set of studies, the metastasis suppressor, N-myc downstream regulator gene 1 (NDRG1) was shown to inhibit basal and hypoxia-induced autophagy via a dual-inhibitory mechanism involving impaired autophagic degradation and autolysosome formation in PDAC cells [49,310]. This inhibitory effect of NDRG1 on autophagy was shown to be mediated by suppression of PERK-eIF2α pathway [310]. Furthermore, NDRG1-mediated suppression via the PERK-eIF2α pathway was found to reduce migration [311]. Collectively, these studies demonstrate that upregulated autophagy in stressed PDAC is a metastasis promoter due to the targeted degradation of crucial proteins required to maintain cell to cell contact and upregulation of EMT marker levels.

With the majority of PDAC patients exhibiting *KRAS* mutations [312], its relationship with the autophagic sequestering protein, p62, is also considered to support metastasis and is highly associated with poor prognosis [313,314]. The recorded high levels of p62 in PDAC can be attributed to the KRAS activation of NF-κB, which transcriptionally induces gene encoding SQSTM1 to produce p62 [315]. p62 was also found to maintain NF-κB activity through a feedforward loop [315]. As NF-κB transcriptional activity is vital for tumor invasion, EMT and anti-apoptosis [316,317], the study by Ling et al. implicates p62, and subsequently autophagy, as a major promoter of metastasis [315].

Another important feature of PDAC is the presence of cancer stem cells (CSCs). CSCs are characterized by their unique properties of self-renewal, sphere forming capacity and de-differentiation states, which contributes to and serves as a basis to cancer metastasis [318,319]. Rausch et al. showed that higher levels of CSC markers correlated with upregulated autophagy in PDAC [320]. Interestingly, autophagy inhibition in pancreatic CSCs resulted in apoptotic cell death and a reduction in migration and tumorigenicity [320]. Hypoxia is a crucial component of autophagic activation, metastasis and supports invasive stem cell-like features in PDAC cell lines [233,321]. Notably, CD133+ pancreatic CSCs were found to be colocalized to the hypoxic region within PDAC tumors [233]. Another study by Yang et al. further supported this hypothesis by positively correlating LC3 expression with the expression of CSC markers, aldehyde dehydrogenase 1 (ALDH1), CD44 and CD133 in PDAC tissues [322]. High co-expression of LC3/ALDH1 was associated with both poor overall survival and progression-free survival [322]. Indeed, the inhibition of autophagy by silencing *ATG5*, *ATG7* and *BECN1* or the administration of CQ significantly reduced pancreatic CSC population and activity [322]. These results suggest that stress-induced autophagy supports metastasis through the sustenance of pancreatic CSCs.

#### 5.3.2. Autophagy as a Metastasis Suppressor in Pancreatic Cancer

Where the previous studies demonstrate autophagy as a metastasis promoter, there are also studies that suggest an opposing effect. For instance, Akar et al. found that the elevated expression of the tissue transglutaminase, TG2, has been implicated in increased drug resistance, supporting metastatic phenotypes and poor patient prognosis in PDAC [323]. More specifically, TG2 increases EMT markers (vimentin, N-cadherin and fibronectin) and decreases E-cadherin levels [323]. The inhibition of protein kinase C-delta (PKCδ), which is vital for TG2 expression, resulted in excessive autophagic activation and Beclin-1-mediated cell death [323]. This result indicates that TG2-mediated autophagy suppression supports metastasis and implicates that autophagic activity suppresses metastasis.

Studies demonstrating autophagic interactions that the partial (heterozygous deletion) or complete (homozygous deletion) loss of certain autophagy genes, have been shown to lead to contrasting outcomes. For instance, ATG5, a crucial protein in autophagosome formation, appears to contribute to metastatic capabilities in PDAC. Notably, there was a clear phenotypic difference between the complete and partial loss of *ATG5* in autophagy-proficient transgenic mice with *KRAS^G12D^* PDAC [324]. The homozygous knockout of *ATG5* in mice harboring *KRAS^G12D^* supported tumor initiation but prevented PDAC tumors from progressing into more malignant states [324]. Whereas, the heterozygous knockout of *ATG5* in the same mouse model increased tumor incidence, malignancy and metastatic potential in PDAC by enhancing neoplastic migration and invasion when compared to the homozygous *ATG* knockout or *KRAS^G12D^* control mice [324]. This relationship could be attributed to the numerous non-canonical autophagy-associated and intracellular degradation pathways that are responsible for the compensatory switch for the loss of ATG5, or as a protective mechanism exerted by PDAC cells. Therefore, this study demonstrates that partial loss of autophagy led a highly metastatic phenotype compared to mice with completely deficient or proficient autophagic activity.

Collectively, the different models used in these studies suggest that autophagy plays both a pro- and anti-metastatic role in PDAC. This is presumably due to the diverse role of the molecules and proteins involved in autophagic regulation and thus, indicates that these interactions require careful consideration throughout the development of PDAC chemotherapeutic strategies that involve the autophagic pathway.

## 6. Inhibiting Autophagic Machinery

As autophagy is a dynamic and sequential pathway, it is possible to induce therapeutic inhibition at either the initiation or degradation stage to achieve reduced autophagic flux.

### 6.1. Targeting Late-Stage Autophagy

**Figure 5:**
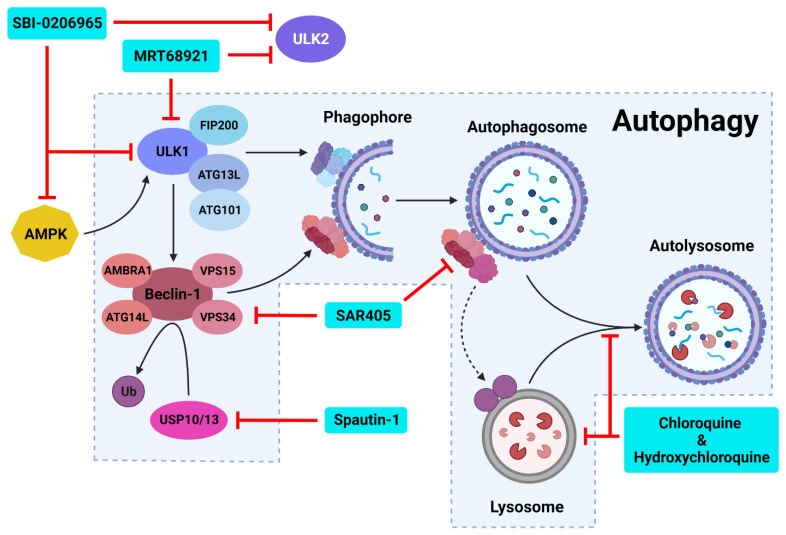
**Autophagy Inhibitors Targeting Different Arms of the Autophagic Pathway.** SBI-0206965 inhibits AMPK, ULK1 and ULK2 to prevent autophagic initiation. MRT68921 inhibits ULK1 and ULK2 to prevent autophagic initiation. SAR405 inhibits VPS34 on the PI3KC-C1 and PI3KC-C3 to suppress initiation and fusion stages. Spautin-1 inhibits USP10 and USP13 to promote UPS-mediated degradation of Beclin-1. CQ/HCQ inhibit the lysosomal activity and fusion with autophagosomes. *Created with BioRender.com (accessed on 27 January 2022)*.

The first autophagic inhibitors, i.e., chloroquine (CQ) and its derivatives, targeted the integrity of the lysosomes and autophagosome fusion stage [82,83]. The association between CQ and cancer has expanded from its effectiveness in malaria and Burkitt’s Lymphoma [325,326]. As an alkaline compound, the mechanism of action involved lysosomal interference by raising the pH to render the acidic hydrolases ineffective [325,327]. More recently, researchers identified that its therapeutic properties also involves lysosomal membrane permeabilization and impaired lysosomes, in addition to raising the lysosomal pH (Figure 5) [328]. This results in autophagy interference since the autophagosomes and lysosomes are unable to fuse and complete the degradation of the targeted cellular content [78,329]. Although CQ was passed through clinical trials for its use as an autophagy inhibitor against cancer, long term users suffered undesirable side effects and it was modified into hydroxychloroquine (HCQ), which is a safer and more efficacious analog [329]. Currently, CQ and HCQ have been approved by the FDA for rheumatoid arthritis, malaria and assessed in a range of clinical trials involving cancer treatment [330,331]. Interestingly, there is a recent report of a novel, more efficacious analog, EAD1, capable of increasing the sensitivity of PDAC neoplasms and stem cells to radiation therapy [332]. Additionally, EAD1 was shown to be more efficacious in *KRAS^G12D^* mutant cell lines, indicating its potential as a treatment option for PDAC [332].

The study by Wolpin et al. demonstrated that HCQ as a monotherapy against chemo-resistant PDAC patients was inconsistent and inefficacious [333]. As most PDAC patients are diagnosed at an advanced stage and may be chemo-resistant [121], it is understandable that an autophagy inhibitor is unlikely to be highly effective as a standalone treatment. However, since aggressive tumors such as PDAC rely heavily on autophagy as a survival mechanism [210], the use of CQ remains promising as a combination therapy. In fact, a recent study modified drug delivery using a pH-sensitive nano-bomb containing CQ and gemcitabine that provides a deeper penetration into the resistant PDAC stroma [246]. This combination was highly effective at reducing tumor growth and metastasis through: (1) autophagic inhibition; (2) cytotoxic gemcitabine effects; and (3) the downregulation of MMP-2 to reduce ECM modification and density [246]. The innovation of this drug delivery system provides a more effective therapeutic strategy that can be used to enhance current compounds that lack tissue penetration.

An interesting study by Bryant et al. showed that *KRAS* knockdown, an ERK inhibitor (SCH772984) and a MEK inhibitor (binimetinib) led to an increased autophagic flux in PDAC cells [291]. These results indicated that upregulated autophagy could be a primary survival mechanism in the PDAC cells in response to KRAS/MAPK pathway inhibition [291]. This was supported by the observed therapeutic synergy and an effective anti-tumor response upon the administration of MEK/ERK inhibitors with HCQ [291]. More recently, the same lab researchers further explored this synergy. Using gene set enrichment analysis, they identified that IGF1R loss was a sensitizer for CQ treatment [334]. Similarly to the previous study, IGF1R inhibition was used to stimulate autophagic flux, which enhanced the effect of autophagy inhibition by CQ in 3D spheroid models [334]. Both of these results reinforce the alternate avenue of increase tumor dependance on autophagy and then blocking it by potent autophagy inhibitors.

Due to the importance of the MAPK pathway, specifically ERK1/2, on promoting EMT and tumor growth, a recent study further supported this work and observed a similar effect. The combination of ERK inhibitor (SCH772984) and CQ demonstrated significant effectiveness at reducing PDAC cell viability and inducing apoptosis [258]. Furthermore, this combination treatment in xenografted mouse models led to the suppression of PSC-mediated fibrosis, induction of PSC senescence and reduction in metastatic potential [258].

Notably, a randomized phase II preoperative 2020 study by Zeh et al. compared the efficacy of chemotherapy (gemcitabine/nab-paclitaxel) in the presence or absence of autophagy inhibition (HCQ) in PDAC [179]. Patients treated with chemotherapy and HCQ demonstrated significant autophagic inhibition, immune cell infiltration and improved pathological response compared to patients treated with chemotherapy alone [179]. Gemcitabine treatment has been found to induce autophagy though the mTORC1/AMPK pathways upstream of autophagic initiation [295], and thus, could potentially explain the observed synergy with autophagy inhibitors. Furthermore, a recent phase I/II clinical trial involving patients with advanced PDAC were treated with a neoadjuvant combination of HCQ and gemcitabine prior to resection [335]. The median overall survival was 31 months and 31% of the patients who received a pancreaticoduodenectomy survived after 5 years, which is a significant improvement when compared to current PDAC survival rates (discussed in Section 3.1) [336]. There are currently four active clinical trial studies on autophagy and PDAC, all of which include an established chemotherapeutic and HCQ as combination therapy (Table 1).

### 6.2. Targeting Autophagy Initiation

Increased understanding of the initiation sequence that involves mTORC1, AMPK, ULK1 and PI3KC3-C1 complexes have led to the development of more modern autophagy inhibitors [60,93,335]. Targeting the upstream proteins and pathways (e.g., mTORC1 or AMPK) that regulate the activation of autophagy could be a viable option. However, in complex neoplasms, this typically results in unwanted side effects or non-specific activity. The core proteins involved in the autophagic initiation stage can be narrowed down to the activation of both the ULK1 and PI3KC3-C1 complexes, which could provide more specific targeting of the autophagy pathway [57,62,93]. While the autophagic initiation inhibitors seem promising and may encourage further PDAC research, none have yet entered clinical trials as an anti-cancer agent.

#### 6.2.1. ULK1 Complex Inhibitors

##### MRT68921

One of the first specific ULK1/2 inhibitors, MRT68921, was identified as a more potent edition of its predecessor MRT67307 [337]. MRT68921 showed significant ULK1 and ULK2 inhibition resulting in a markedly suppressed rate of ULK1 kinase activity and reduced autophagic flux under both nutrient starved and sufficient conditions (Figure 5) [337]. Interestingly, in ovarian cancer spheroids, MRT68921 was able to block autophagic flux and effectively kill tumor cells [338]. Recently, Chen et al. examined MRT68921 cytotoxicity in a broad range of cancer cells and healthy cells [339]. They identified that as a single agent, MRT68921 achieved potent and selective anti-proliferative activity against cancer cells [340]. This was found partly due to ROS induction and increased apoptosis. The *in vivo* studies found reduced tumor size, cell migration and apoptosis [340].

Currently, there are limited studies assessing the effect of MRT68921 in PDAC models. Notably, a recent study identified that MRT68921 increased macropinosomes and reduced autophagosome levels in PDAC [339]. This result represents a switch from autophagy to macropinocytosis, a process involving the internalization of extracellular nutrients, as another upregulated survival pathway in PDAC [339]. In allografted mouse models, MRT68921 was found to inhibit LC3-II formation and limit autophagy but produced a minimal effect on tumor size [339]. MRT68921 was found to be highly synergistic with the macropinocytosis inhibitor, EIPA and demonstrated significant anti-tumor activity [339]. These results suggest that the combination of an autophagy initiation inhibitor and macropinocytosis inhibitor could be key in overcoming the survival mechanisms within PDAC. However, further validation of these studies is warranted.

##### SBI-0206965

A study by Egan et al. demonstrated the inhibitory properties of SBI-0206965 against autophagy [55]. The compound was described as highly selective for ULK1 and ULK2, where it demonstrated significant mTOR-induced autophagic inhibition (Figure 5) [55]. They further assessed the anti-tumor effects of SBI-0206965 in A549 lung cancer cells and demonstrated that SBI-0206965 was only able to induce a significant apoptotic response when used in combination with mTORC1 inhibitors such as rapamycin and AZD8055 [55]. An interesting effect was also observed in renal carcinoma cells where SBI-0206965 cytotoxicity was more effective under starvation conditions [341]. Furthermore, a study involving non-small cell lung cancer showed synergistic activity between SBI-0206965 and cisplatin [342]. Since cisplatin induces both apoptosis and autophagy, the combination with SBI-0206965 was shown to be beneficial as it sensitized the cells to cisplatin-induced apoptosis by suppressing the autophagic survival pathway [342].

In addition to its inhibitory effects on ULK1, SBI-0206965 has been recently identified to inhibit AMPK signaling by up to 40-fold higher and more selectively than the established AMPK inhibitor Compound C (Figure 5) [343]. This was also confirmed in mouse skeletal muscle as SBI-0206965 was able to potently ameliorate AICAR-stimulated glucose transport, which is a well-established AMPK activator [344]. Simultaneous AMPKα2 and ULK1 inhibition provides SBI-0206965 with a unique therapeutic advantage as it works on two major autophagy regulators. Notably, MRT68921 and SBI-0206965 are also shown to exert their cell death mechanisms via the activation of the caspase-3/8 apoptosis pathway and destabilization of Bcl-2 and BclxL [340,342]. SBI-0206965 demonstrated a similar anti-proliferative ability as MRT68921 in a range of cancer cell lines [340].

A more recent study identified a new compound named SBP-7455 that demonstrated a 10-fold increased potency than SBI-0206965 and was more effective at blocking autophagic initiation in triple negative breast cancer [167]. SBP-7455 showed synergy with the PARP inhibitor, olaparib, to significantly reduce cell viability [167]. These results strongly encourage more scientific research into ULK1 inhibitors in PDAC as they exert potent autophagic suppression and have shown synergistic anti-cancer activity with a variety of compounds such as upstream KRAS inhibitors (AMG510) [345], ERK inhibitors (SCH772984) [291], PARP inhibitors (for BRCA mutant tumors) [346] and gemcitabine [295,323,347].

#### 6.2.2. PI3K Class III Complex Inhibitors

##### Spautin-1

In conjunction with the ULK1 complex, autophagy is initiated by the PI3KC3-C1 protein conjugate consisting of a core of Beclin-1, VPS15 and VPS34 subunits [60,63]. As a means of regulation, Beclin-1 is perpetually deubiquitinated by ubiquitin specific peptidase 10 (USP10) and USP13 to prevent proteasomal degradation from the UPS pathway [348]. Spautin-1 is a selective inhibitor for both USP10 and USP13, which leads to Beclin-1 degradation by the UPS and hence, prevents PI3KC3-C1 formation resulting in the suppression of autophagic initiation (Figure 5) [348].

Spautin-1 was shown to significantly reduce cell viability in selected cervical cancer cells (BCaP-37) and breast cancer cells (MCF-7 and BT549) under glucose starvation conditions [348]. Interestingly, USP10 is also a deubiquitinating agent for p53 [349] and nuclear p53 can induce the transcription of autophagy related genes such as *ULK1* and *ATG7* [143,146]. This indicates that Spautin-1 features a potential secondary benefit in *TP53* mutant PDAC. The relationship between p53, apoptosis, autophagy and tumor survival is complex and varies between every neoplasm [146,350]. Of note, Spautin-1 induced p53 degradation may reduce p53-mediated apoptosis and potentially allow for further tumor survival.

Further research involving Spautin-1 in other cancer cell lines such as chronic myeloid leukemia had demonstrated an apoptotic effect via GSK3β activation and showed that Spautin-1 was capable of sensitizing the cells to imatinib chemotherapy [351]. Spautin-1 synergized with doxorubicin in canine osteosarcoma to significantly increase cell death and colony formation [352] and reduced metastasis in hepatocellular carcinoma [353]. Currently, the published research on Spautin-1 in the pancreas is limited to acute pancreatitis and involves the inhibition of impaired autophagy [354]. A recent study investigating the combination of MAPK and autophagy inhibition utilized Spautin-1 in PDAC [290]. As the ERK inhibitor, SCH772984, suppressed the pro-survival activity of ERK1/2, it also stimulated autophagy. Spautin-1 was administered in combination with SCH772984 to oppose this and inhibit the increased autophagy. This led to significantly reduced cell viability in PDAC cell lines, suggesting a promising utility for Spautin-1 in PDAC [290].

Another interesting study involving PDAC assessed the effects of Spautin-1 with niclosamide, a compound known to inhibit cellular metabolism and induce apoptosis [355]. PDAC cells pre-incubated with Spautin-1 and subsequently treated with niclosamide resulted in markedly reduced apoptotic cell death [355]. Spautin-1 was found to reduce Beclin-1 levels which enabled abundant Bcl-2 to prevent BAX/BAK oligomerization and subsequently, reduced apoptosis [355]. Notably, these anti-apoptotic effects from Spautin-1 need to be considered in apoptosis-induced PDAC therapy.

##### SAR405

SAR405 is a novel VPS34 inhibitor, which is selective for PI3KC3-C1/C2 and features some inhibition of PI3KC1 and p-AKT at higher concentrations (Figure 5) [356]. Using fluorescence microscopy, SAR405 was shown to prevent autophagosome formation and late endosome to lysosomal formation [357]. Thus, SAR405 has a dual point of autophagy inhibition at both the initiation and late stages (Figure 5). This is potentially due to the involvement of VPS34 in autophagy initiation (PI3KC-C1) and influence in autophagosome and lysosome fusion when bound to additional proteins UVRAG and Rubicon (PI3KC-C2) [60,62].

SAR405 was shown to synergize with HER2 inhibitors in HER2+ breast cancer [358]; celecoxib in osteosarcoma [359]; and cisplatin in urothelial carcinoma [359]. This synergism led to significant inhibition of autophagic flux, leading to an increase in apoptosis induction. Additionally, SAR405 was able to decrease tumor growth and promote inflammation in melanoma and colorectal cancer cells to enhance anti–PD-L1/PD-1 immunotherapy [360]. This result could be associated with the involvement of autophagy and immune evasion mediated by the degradation of MHC-I molecules [287].

*TSC* knockout mouse embryonic fibroblasts were observed to respond to autophagy deficiency through the upregulation of macropinocytosis, a survival pathway that internalizes proteins for lysosomal degradation [361]. This study demonstrated that VPS34, the target of SAR405, was a major regulator supporting macropinocytosis. Therefore, SAR405-mediated VPS34 inhibition was demonstrated to be effective at suppressing both autophagy and macropinocytosis [361]. Notably, these results are in contrast to the study by Su et al. [339], which demonstrated a switch to macropinocytosis upon autophagy inhibition by MRT68921. This is shown to be highly beneficial as an anti-tumor property of SAR405, as macropinocytosis is a critical process whereby cells can obtain extra cellular nutrition and can sustain tumor survival. Currently, there are no studies assessing the effects of SAR405 in PDAC models and would be an interesting area for future research.

#### 6.2.3. New Autophagy Initiation Inhibitors

Even though inhibitors of ULK1 or PI3KC3 complexes are yet to enter PDAC clinical trials, there are more compounds being developed that await thorough *in vitro* evaluation. A recent study has screened the Published Kinase Inhibitor Set by Glaxo-Smith-Kline resulting in the identification of two ULK1 inhibitors (GW837331X and GW406108X) and two VPS34 inhibitors (GSK2358994A and GW429374A) [362]. Both sets of compounds were able to effectively inhibit their targets activity and subsequently prevent autophagic flux [362]. However, GW837331X and GW406108X were found to be less potent than MRT68921 and SBI0206965 [362].

### 6.3. Natural Products Targeting Autophagy

In addition to synthetic small molecule agents, natural products and their derivatives have also been shown to suppress PC tumour progression via interactions with the autophagy pathway. The mechanism of action of these natural products is typically not well elucidated but does provide an understanding that various compounds can beneficially interact with autophagy in different ways. One of the common avenues natural products achieve this is through the dysregulation of apoptosis pathways (namely through Bcl-2 and caspases) to achieve cell death.

A proteoglycan extracted from *Ganoderma lucidum* known as Fudan-Yueyang-Ganoderma lucidum (FYGL) was found to increase autophagosome levels and prevent autolysosome fusion, indicating late-stage autophagic inhibition effects [363]. FYGL also reduced Bcl-2 expression and increased cleaved caspase-3 which led to increased ROS and reduced MMP, indicating apoptotic potential [363]. However, these results were only present in PANC-1 and not MiaPaCa2 cells, suggesting cell type specific effects and require further investigations. Similarly to FYGL, alantolactone also induced apoptosis by increasing cleaved caspase-3 and impaired autophagic degradation which led to autophagosome accumulation in PDAC cells [364]. When used in combination with *ATG5* knockdown, the researchers observed a reduced effectiveness and concluded that alantolactone toxicity was dependent on accumulated autophagosomes [364]. Alantolactone is also shown to have a significant synergy with oxaliplatin resulting in enhanced cytotoxicity in PDAC cells [364]. Additional combination studies have demonstrated that alantolactone suppresses STAT-3, and subsequently Bcl-2 activity, in PDAC and have a remarkable synergy with epidermal growth factor (EGFR) inhibitors such as erlotinib and afatinib [365,366].

Curcumin is another natural compound which mediates its anti-tumor effects by increasing the ratio of BAX to Bcl-2, leading to increased rates of apoptosis [367]. The relationship between curcumin and autophagy depends on the reduced Bcl-2 levels as it promotes increased Beclin-1-mediated autophagic initiation [367]. Therefore, curcumin differs to the previous compounds as it lacks any other direct inhibitory effects on autophagy and results in the overall upregulation of autophagy and apoptosis. Interestingly, curcumin was shown to increase PDAC chemo-sensitivity to gemcitabine [368]. The fact that alantolactone and curcumin both mediate the same mechanism of action on BAX/Bcl-2 and beneficially synergizes other cytotoxic chemotherapeutics, yet mediate contrasting effects on autophagy, suggests that these compounds have more unknown cellular interactions or that they exhibit low potency for BAX/Bcl-2. Such non-specific features are less desirable than targeted synthetic compounds involved in apoptosis and autophagy-mediated therapeutics.

Ursolic acid (UA) is a natural triterpene, which is known to increase cellular stress. In PDAC cells, the increased stress upregulated autophagy, while the reduced RAGE expression was shown to induce cell cycle arrest and apoptosis [369]. RAGE is known as a mediator between apoptosis and autophagy where it promotes tumor survival through autophagy activation and anti-apoptotic effects that involve p53 dephosphorylation and increased Bcl-2 expression [370]. This observation led to the assessment of combining UA with chemotherapeutics, such as gemcitabine [369]. Therefore, as UA inhibits RAGE the tumor cells become more sensitive to cytotoxic chemotherapeutics. The marine sponge extract scalarin also reduces RAGE levels and induces apoptosis in PDAC cells [371]. In contrast to UA, scalarin was shown to inhibit stress-induced autophagy through LC3-II accumulation [371]. The fact that UA and scalarin both mediate the same mechanism of action on RAGE yet have opposing effects on autophagy suggests that these compounds have more unknown cellular interactions or that RAGE is not an ideal target for apoptosis and autophagy-mediated therapeutics.

Notably, periplocin has been found to induce caspase-dependent apoptosis through AMPK activation/mTOR inhibition [372]. However, because of this, periplocin also induced excessive autophagy activation and autophagy-dependent cell death [372]. This effect indicates that natural products may be useful in combination treatments with other cytotoxic compounds but lack potent specificity to target desirable proteins and, therefore, demonstrate a wider range of pathway promiscuity that may counter or limit the desired effect. It is becoming more established that autophagic activation suppresses PC tumour initiation while reduced autophagic activity is more effective at reducing PC tumor progression [304,373]. Considering this, the novel concept of inducing cytoprotective autophagy from either a synthetic compound or natural product to be combined with strong and targeted autophagic inhibition has been explored. This technique utilizing autophagic reliance was used with the combination of fisetin, 3-MA and chloroquine [374]. Fisetin was found to stimulate apoptosis and cytoprotective autophagy through the ER and mitochondrial stress pathways [374,375]. This protective autophagy was then blocked by 3-MA and chloroquine to produce a markedly decreased cell viability when compared to fisetin alone [374].

Overall, these studies suggests that while the use of natural products in autophagy-related therapy remains a promising avenue for treatment, their limited efficacy and potency and promiscuous targeting must be taken into consideration when compared to targeted synthetic small molecule inhibitors of autophagy related proteins.

## 7. Conclusions

This review of current literature demonstrates a critical role of the autophagic pathway in key mechanisms involved in PDAC progression, such as metabolic reprogramming, immune evasion, anti-apoptosis, TME-induced stress, metastasis, perineural invasion, etc. Importantly, there is a new wave of next generation autophagy inhibitors which have shown potential to be developed as novel therapeutics for the treatment of PDAC. With poor patient outcomes after PDAC chemotherapy, the development of these promising compounds can be clinically applied to significantly improve PDAC patient survival and increase quality of life. Due to modest improvement in therapeutic outcomes using current treatments (e.g., FOLFIRINOX, gemcitabine/abraxane), autophagy inhibitors could be implemented into the current clinical approach to PDAC treatment. Therefore, further research to comprehensively understand the role of autophagy in PDAC progression and development of autophagy inhibition based anti-PDAC therapeutic strategies are highly warranted.

## Figures and Tables

**Table 1:** Current clinical trials using chloroquine or hydroxychloroquine to treat PDAC. (Source: clinicaltrial.gov, accessed on 28 June 2022).

Type of PC	Study Design	Drug and Dose	Status	Serial No.
Metastatic PDAC	Phase 2, Non-randomized, Open label	400 mg HCQ OR 600 mg HCQ, BID for 4 weeks	Completed	NCT01273805
Inoperable locally advanced and metastatic PC	Phase 1, Open label,	mFOLFIRINOX (backbone) + 250 mg chlorphenesin carbamate + 200 mg HCQ, BID for 48 weeks	Recruiting	NCT05083780
Resectable PC	Phase 2, Open label	Photon/proton radiation during week 2 for 5 days + 825 mg/m^2^ capecitabine BID for 10 days + 400 mg HCQ BID from day 1 until surgery	Active not recruiting	NCT01494155
Metastatic PDAC, stage IV PC	Phase 1 pilot, Open label,	Binimetinib + HCQ, BID for 2 weeks	Recruiting	NCT04132505
Advanced PDAC, metastatic PDAC, stage IV PC	Phase 2, Open label	Paricalcitol three times weekly + HCQ BID + gemcitabine weekly + nab-paclitaxel 30 min weekly	Recruiting	NCT04524702
Metastatic PDAC, Stage II, Stage IIA, Stage IIB, Stage III, Stage IV PC, Unresectable PDAC	Phase 1, Open label	Trametinib QD + HCQ QD or BID for 4 weeks	Recruiting	NCT03825289
Advanced PDAC Metastatic PDAC	Phase 1/2, Randomized, Open label	Gemcitabine 1000 mg/m^2^ weekly + nab-paclitaxel 125 mg/m^2^ weekly OR gemcitabine 1000 mg/m^2^ weekly + nab-paclitaxel 125 mg/m^2^ weekly + HCQ 600 mg/m^2^ BID, for 15 days	Completed	NCT01506973
Pancreatic cancer	Phase 2, Randomized, Open label	Gemcitabine 1000 mg/m^2^ + nab-paclitaxel 125 mg/m^2^, weekly for 45 days OR gemcitabine 1000 mg/m^2^ + nab-paclitaxel 125 mg/m, weekly for 45 days + HCQ 600 mg/m^2^ QD or BID until surgery	Completed	NCT01978184
Pancreatic cancer	Phase 1/2, Open label	Gemcitabine 10 mg/m^2^/min (dependent on dose) on day 1 and 15 + HCQ 200, 400, 600, 800, 1000, or 1200 mg BID for 31 days	Completed	NCT01128296

## Abbreviations

PC: pancreatic cancer; PDAC: pancreatic ductal adenocarcinoma; KRAS: Kirsten rat sarcoma viral oncogene homologue; TP53: tumor protein p53; TME: tumor microenvironment; UPS: ubiquitin-proteasomal system; CMA: chaperone-mediate autophagy; Hsc70: heat shock cognate protein of 70 kDa; p62: Sequestosome-1; ULK1: UNC-51-like kinase 1; PI3K: phosphodioniside 3-kinase; PI3KC3-C1/2: PI3K class III complex 1/2; FIP200: family interacting protein 200 kDa; ATG: autophagy gene/protein; BNIP-3: BCL2/adenovirus E1B 19 kDa protein-interacting protein 3; VPS: vacuolar protein sorting; AMBRA1: activating molecule in Beclin1-regulated autophagy; ER: endoplasmic reticulum; UVRAG: UV radiation resistance-associated gene protein; BNIP3: BCL2/adenovirus E1B 19 kDa protein-interacting protein 3; LC3/MAP1LC3: microtubule-associated protein 1A/1B-light chain 3; LC3-II: LC3-phosphatidylethanolamine conjugate; PE: phosphatidylethanolamine; GABARAP: Gamma-aminobutyric acid receptor-associated protein; GATE-16: Golgi-associated ATPase Enhancer of 16 kDa; BNIP3L: BCL2/adenovirus E1B 19 kDa protein-interacting protein 3-like; NBR1: Neighbour of BRCA1 gene 1 protein; Alfy: autophagy-linked FYVE protein; GTPase: guanosine triphosphate hydrolase enzyme; Rab: Ras-related protein; Rubicon: RUN domain and cysteine-rich domain containing, Beclin-1-interacting protein; AKT: protein kinase B; mTORC1: mammalian target of rapamycin complex 1; MAPK: mitogen activated protein kinase; AMPK: adenosine monophosphate-activated protein kinase; Bcl-2: B-cell lymphoma 2; PIP2: phosphatidylinositol (3,4,5)-bisphosphate; PIP3: phosphatidylinositol (3,4,5)-trisphosphate; PTEN: phosphatase and tensin homolog; TSC1/2: tuberous sclerosis complex 1/2; RHEB: Ras homolog enriched in brain; GβL: target of rapamycin complex subunit LST8; PRAS40: proline-rich AKT substrate of 40 kDa; Raptor: regulatory-associated protein of mTOR; S6K1: ribosomal protein S6 kinase beta-1; eIF-4E: eukaryotic translation initiation factor 4E; 4E-BP1: Eukaryotic translation initiation factor 4E-binding protein 1; RAF: rapid accelerated fibrosarcoma; MEK: mitogen-activated protein kinase kinase; ERK: extracellular signal-regulated kinase; ATP: adenosine triphosphate; AMP: adenosine monophosphate; ADP: adenosine diphosphate; LKB1: liver kinase B1; CaMKKß: Ca^2+^/calmodulin-dependent kinase kinase β; TGF-β: transforming growth factor-β; TAK1: transforming growth factor-β-activated kinase 1; BH3: Bcl-2 Homology 3; BAX: Bcl-2-associated X protein; BAK: Bcl-2 homologous antagonist killer; JNK1: c-Jun N-terminal kinase 1; Bad: BCL2 associated agonist of cell death; Noxa: phorbol-12-myristate-13-acetate-induced protein 1; Puma: p53 upregulated modulator of apoptosis; FOLFIRINOX: leucovorin calcium, fluorouracil, irinotecan hydrochloride, and oxaliplatin; SMAD4: SMAD Family Member 4; CDKN2A: cyclin dependent kinase inhibitor 2A; HRAS: Harvey rat sarcoma virus; NRAS: neuroblastoma RAS viral oncogene homolog; GAP: GTPase activating protein; EGF: epithelial growth factor; IGF: insulin-like growth factor; CAF: cancer-associated fibroblast; NFĸB: nuclear factor-ĸ-B; SMAD-4: SMAD family member 4; BRCA: breast cancer gene; Mdm2: mouse double minute 2 homolog; CAF: cancer-associated fibroblast; PARP: poly-ADP ribose polymerase; ROS: reactive oxygen species; TCA: tricarboxylic acid; NOX: nicotinamide adenine dinucleotide phosphate oxidase; HIF-1: hypoxia inducible factor 1; BRCA: breast cancer type 1 susceptibility protein EMT: epithelial-mesenchymal transition; Snail: zinc finger protein SNAI1; Slug: zinc finger protein SNAI2; Twist: Twist-related protein 1; ZEB: zinc finger E-box binding homeobox; NHE-1: sodium-hydrogen exchanger isoform-1; v-ATPase: pH: potential of hydrogen; ASIC: acid-sensing ion channels; PSC: pancreatic stellate cells; VEGF: vascular endothelial growth factor; PDGF: platelet-derived growth factor; ECM: extracellular matrix; E-cadherin: epithelial cadherin; ITGAV: integrin αV; 3-MA: 3-methyladenine; FAK: focal adhesion kinase; SHH: sonic hedgehog; COX-2: cyclooxygenase-2; NGF: nerve growth factor; TNF-α/β: tumor necrosis factor alpha/beta; IL: interleukin; TRAIL: tumor necrosis factor-related apoptosis-inducing ligand; GATA1: GATA-binding factor 1; acetyl-CoA: acetyl coenzyme A; MMP: matrix metalloproteinase; TIMP: tissue inhibitor of metalloproteinase; YAP-1: yes-associated protein 1; EPC: endothelial progenitor cell; Th: T-helper; M1: classically activated macrophage; M2: alternatively activated macrophage; JAK-STAT: Janus kinase-signal transducer and activator of transcription; MHC-I: major histocompatibility complex class I; EAD1: effector associated domain 1; EIPA: 5-(N-ethyl-N-isopropyl)-Amiloride; CQ: chloroquine; HCQ: hydroxychloroquine; AICAR: 5-aminoimidazole-4-carboxamide-1-β-D-ribofuranoside; BclxL: B-cell lymphoma-extra large; USP: ubiquitin specific peptidase; IGF1R: insulin-like growth factor 1 receptor; GSK3β: glycogen synthase kinase 3 beta; HER2: human epidermal growth factor receptor 2; PD-1: programmed cell death protein 1.
